# Dietary intervention improves health metrics and life expectancy of the genetically obese Titan mouse

**DOI:** 10.1038/s42003-022-03339-3

**Published:** 2022-05-03

**Authors:** Annika Müller-Eigner, Adrián Sanz-Moreno, Irene de-Diego, Anuroop Venkateswaran Venkatasubramani, Martina Langhammer, Raffaele Gerlini, Birgit Rathkolb, Antonio Aguilar-Pimentel, Tanja Klein-Rodewald, Julia Calzada-Wack, Lore Becker, Sergio Palma-Vera, Benedikt Gille, Ignasi Forne, Axel Imhof, Chen Meng, Christina Ludwig, Franziska Koch, John T. Heiker, Angela Kuhla, Vanessa Caton, Julia Brenmoehl, Henry Reyer, Jennifer Schoen, Helmut Fuchs, Valerie Gailus-Durner, Andreas Hoeflich, Martin Hrabe de Angelis, Shahaf Peleg

**Affiliations:** 1 Research Group Epigenetics, Metabolism and Longevity, Research Institute for Farm Animal Biology (FBN), 18196 Dummerstorf, Germany; 2 Institute of Experimental Genetics, German Mouse Clinic, Helmholtz Zentrum München, German Research Center for Environment and Health (GmbH), 85764 Neuherberg, Germany; 3 Department of Molecular Biology, Biomedical Center Munich, Ludwig-Maximilians University, 82152 Planegg-Martinsried, Germany; 4 Institute Genetics and Biometry, Lab Animal Facility, Research Institute for Farm Animal Biology (FBN), 18196 Dummerstorf, Germany; 5 German Center for Diabetes Research (DZD), 85764 Neuherberg, Germany; 6 Institute of Molecular Animal Breeding and Biotechnology, Gene Center, Ludwig-Maximilians-University Munich, 81377 Munich, Germany; 7 Institute of Reproductive Biology, Research Institute for Farm Animal Biology (FBN), 18196 Dummerstorf, Germany; 8 Bavarian Center for Biomolecular Mass Spectrometry (BayBioMS), Technical University of Munich, 85354 Freising, Germany; 9 Institute of Nutritional Physiology, Research Institute for Farm Animal Biology (FBN), 18196 Dummerstorf, Germany; 10 Helmholtz Institute for Metabolic, Obesity and Vascular Research (HI-MAG) of the Helmholtz Zentrum München at the University of Leipzig and University Hospital Leipzig, Leipzig, Germany; 11 Institute for Experimental Surgery, Rostock University Medical Center, Rostock, Germany; 12 Institute for Genome Biology, Research Institute for Farm Animal Biology (FBN), 18196 Dummerstorf, Germany; 13 Department of Reproduction Biology, Leibniz Institute for Zoo and Wildlife Research (IZW), Berlin, Germany; 14 Chair of Experimental Genetics, TUM School of Life Sciences (SoLS), Technische Universität München, 85354 Freising, Germany; 15 Institute of Neuroregeneration and Neurorehabilitation of Qingdao University, Qingdao, China

**Keywords:** Molecular biology, Biochemistry, Physiology

## Abstract

Suitable animal models are essential for translational research, especially in the case of complex, multifactorial conditions, such as obesity. The non-inbred mouse (*Mus musculus*) line Titan, also known as DU6, is one of the world’s longest selection experiments for high body mass and was previously described as a model for metabolic healthy (benign) obesity. The present study further characterizes the geno- and phenotypes of this non-inbred mouse line and tests its suitability as an interventional obesity model. In contrast to previous findings, our data suggest that Titan mice are metabolically unhealthy obese and short-lived. Line-specific patterns of genetic invariability are in accordance with observed phenotypic traits. Titan mice also show modifications in the liver transcriptome, proteome, and epigenome linked to metabolic (dys)regulations. Importantly, dietary intervention partially reversed the metabolic phenotype in Titan mice and significantly extended their life expectancy. Therefore, the Titan mouse line is a valuable resource for translational and interventional obesity research.

## Abstract (web summary)

This study further characterizes the non-inbred Titan (also known as DU6) mouse line, which could be a useful model for obesity research.

## Introduction

The identification of suitable animal models is critical for the successful translatability of preclinical data^1–3^. Each animal model presents limitations and the use of complementing models might highlight particular aspects of a disease. This is critical to model complex, multifactorial diseases, such as metabolic disorders and obesity. The genetic homogeneity of inbred lines, for instance, represents both a strength and a limitation for animal models. While they are useful experimentally by limiting the inter-animal variability, inbred lines might not reflect the genetic heterogeneity characterizing some human diseases^1–3^. A model based on the selection of phenotypic traits (e.g., obesity) may provide novel insight into complex disorders^1,4^.

An example of such a selection line is the Dummerstorf “Titan” mouse. Titan is a unique mouse model resulting from an ongoing long-term breeding scheme (currently over 180 generations) that selected mice based on high body mass at six weeks of age^5^. The initial mouse population was created in 1969/1970 by crossing four outbred and four inbred populations^6,7^, resulting in an outbred mouse line (FZTDU) with a polygenetic background. Based on this polygenetic mouse line, the selection for high body mass started in 1975 with every generation consisting of 60–80 mating pairs^8,9^ (see Methods). Importantly, an unselected population was maintained as a control group throughout the selection process^9^.

Several phenotypic and molecular analyses of the Titan mice were conducted at different time points during the ongoing selection experiment^5,10–13^. It was previously reported that 6-week-old Titan mice from earlier selected generations displayed high body mass, as well as elevated levels of fat, insulin, leptin, and growth hormone^5,10,13^. In addition, an epididymal fat gene expression study identified 77 differentially expressed genes (between Titan and control mice) involved in regulatory and metabolic pathways^11^. A more recent study showed that Titan mice (generation 146) displayed impaired glucose tolerance at 6 weeks of age following a high-fat diet, while surprisingly, glucose tolerance progressively improved with age compared to unselected control mice^5^. Within the scope of this observation, the Titan mice have been proposed as a mouse model to specifically study benign obesity^5^. The hallmark of metabolically healthy obesity (MHO) is characterized by preserved insulin sensitivity but further characteristics are important to distinguish between MHO to metabolically unhealthy obesity (MUO)^14^. These include, but are not limited to, lipid profile, liver function, cardiovascular disease, physical activity, and healthy lifespan^14^. Importantly, various obesity-associated comorbidities develop over time and become more prevalent in the later stages of life. Currently, comprehensive analyses of Titan mice throughout their life are lacking^15^.

In this work, we investigate if the obese Titan mice develop typical hallmarks of pathological detrimental obesity (MUO)^14^. To address this hypothesis, we conducted a deep phenotypic characterization of the Titan mice and investigated factors involved in detrimental obesity in humans or other model species such as plasma levels of cholesterol and triglycerides^16^, leptin^16–18^, fibroblast growth factor 21 (FGF21)^19,20^, heart morphology^21^, and alterations of the adipose tissue^22^. Since within a species, body mass and lifespan are two traits known to be inversely correlated to each other^23^, we also assessed whether the lifespan of Titan mice was shortened compared to control mice. In addition, we used recently published genomic sequence data to link several phenotypic features of the mouse line to the specific genetic differentiation caused by the selection procedure^9^.

Since the liver is central to lipid and carbohydrate metabolism, we evaluated possible molecular alterations in this organ. Specifically, we examined transcriptomic^24^, epigenetic and proteomic alterations that might be associated with obesity and diet. Importantly, dietary intervention is a well-studied and robust intervention known to promote a healthy lifespan^25–27^ and improve obesity phenotypes^4,28^ in various animal models. Since not all mouse strains are responsive to caloric restriction^29,30^, we also tested whether the Titan mice might be amenable to therapeutic intervention, using a late and moderate diet intervention as a proof of principle.

Our data support the notion that Titan mice exhibit detrimental obesity (MUO), rather than benign obesity (MHO). Titan mice display genetic variability in regions containing genes associated with several regulatory pathways, notably metabolic. Titan mice exhibit a shorter lifespan, along with molecular changes in the liver. Importantly, dietary intervention in Titan mice partly reversed obesity, transcriptomic, phenotype, and lifespan supporting the idea that Titan mice might be suitable as a translational and interventional model of obesity.

## Results

### Weight development, gross morphology, and abdominal fat distribution

**Fig. 1:**
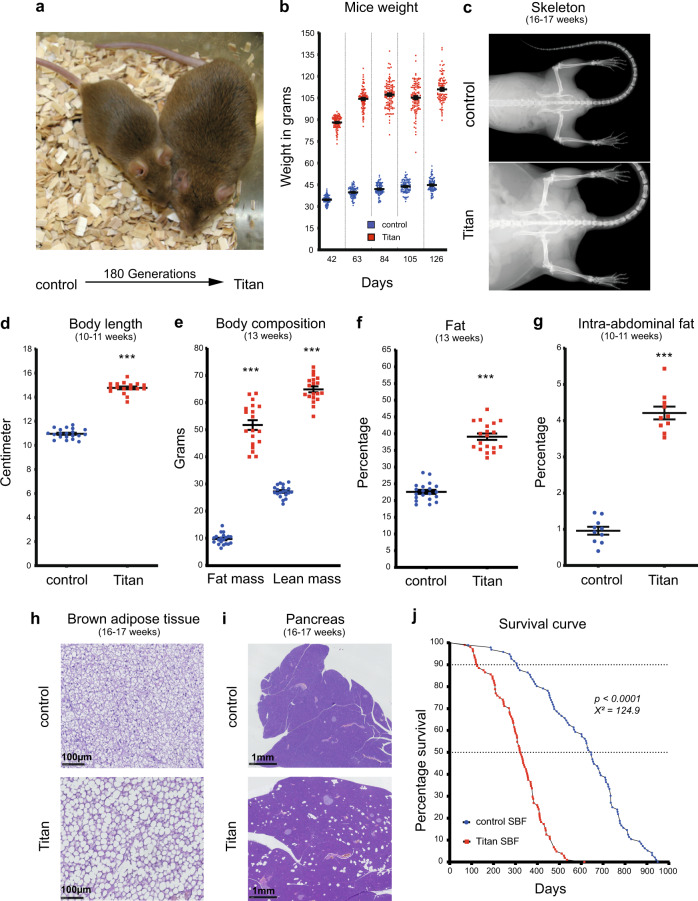
Titan mice, a product of 180 generations of selection, is a severely obese, giant, and short-lived mouse strain. **a** Representative images of unselected control mice (left) and 180th generation-selected Titan mice (right) at 6 weeks of age, the time of the selection. **b** After the first 16–19 weeks of life, Titan mice reached over 110 g on average, whereas average control animals weighed 45 g. **c** Representative X-ray images of control and Titan mice at 16–17 weeks of age. Control (*n* = 38) and Titan (*n* = 31) mice. **d** Body length (cm) of 10–11-week-old Titan mice compared to age-matched control mice. Control (*n* = 20) and Titan (*n* = 17) mice. **e** Minispec data showing total fat and lean mass of control (*n* = 20) and Titan (*n* = 17) mice at the age of 10–11-weeks**. f** Total percentage of fat in control (*n* = 20) and Titan (*n* = 17) mice. **g** Percentage of intra-abdominal fat (*n* = 10 per group) at the age of 10–11-weeks. **h** Representative images of hematoxylin and eosin (H&E)-stained brown adipose tissue from control (*n* = 6) and Titan (*n* = 5) mice (20x magnification) at 16–17 weeks of age. **i** Representative images of H&E-stained pancreas from control and Titan (*n* = 6) mice (2.5x magnification) at 16–17 weeks of age. **j** Comparison of lifespans and mortality rates of Titan and control male mice showing significantly reduced lifespan of Titan mice (log-rank test; *p* < 0.0001, *χ*^2^ = 124.9). ***p < 0.001. Error bars indicate SEM (standard error of the mean). Unpaired two‐tailed *t‐*tests with Welch’s correction were used to calculate *p* values.

We first conducted a thorough characterization of the phenotype in order to support or refute the notion that the obesity in Titan mice is benign (MHO vs. MUO)^5^. We started by revisiting the phenotypic characterization of the male Titan mice, now at generation 180 of the breeding scheme, compared to unselected controls (Fig. 1a). Titan male mice had an average weight of 90 g at the time of selection (6 weeks of age) vs. 35 g for unselected control mice (Fig. 1b). Adult mice further grew to an average of over 110 g (Fig. 1b) and their body and skeleton were substantially larger than those of controls (Fig. 1c). At 10–11 weeks of age, Titan mice reached 14.75 cm in body length (Fig. 1d). Notably, Titan mice exhibited an increase in both total fat and lean mass (Fig. 1e), as well as in fat percentage (Fig. 1f). Fat distribution was also altered, with Titan mice accumulating as much as 4% intra-abdominal fat of total body weight compared to 1% in control mice (Fig. 1g). Overall, our data support the size and obesity phenotypes of Titan mice and also demonstrate an increase in fat mass compared to the previous data^5,10^.

### Hallmarks of detrimental obesity

Since obesity is often linked with hyperlipidemia^16^, we measured the cholesterol and triglyceride plasma levels in Titan and control mice. We detected higher fasting plasma levels of triglycerides, high-density lipoprotein (HDL) and non-HDL cholesterol in Titan compared to control mice at 18–20 weeks of age (Supplementary Fig. 1a–c). Consistent with the notion that hyperlipidemia usually occurs in the presence of insulin resistance, insulin levels were more than 3-fold higher in ad libitum fed 16–17-week-old Titan mice (Supplementary Fig. 1d). Ten-to eleven-week-old Titan mice also show impaired glucose tolerance compared to control mice (Supplementary Fig. 1e). Interestingly, this difference vanished as the mice reached 19–20 weeks of age (Supplementary Fig. 1e).

We also measured leptin levels, a hormone secreted by adipocytes and known to modulate feed intake and fat storage^16–18^. Leptin levels were higher (more than fivefold) in 16–17-week-old Titan than in control mice (Supplementary Fig. 1f). Finally, we found that the plasma concentration of FGF21, a factor associated with metabolic syndrome (MetS) in humans^19,20^, was elevated in the serum (Supplementary Fig. 1g).

As MUO is linked with heart disease, we also assessed histological alterations in the heart of the Titan mice^14,21^. Using Sirius Red staining, we detected fibrotic tissue in the hearts of three out of six 16–17-week-old and five out of six 24–26-week-old Titan mice (Supplementary Fig. 2). Furthermore, whitening of brown adipose tissue (BAT) has been associated with obesity and obesity-related diseases^22^. Indeed, at 16–17 weeks of age, Titan mice showed a substantial whitening with increased lipid droplet sizes in BAT, not observed in control animals (Fig. 1h). Moreover, the perigonadal white adipose tissue (WAT) adipocytes in 16–17-week-old Titan mice were clearly hypertrophic when compared to age-matched controls (Supplementary Fig. 3), in line with the increased size of Titan animals. Despite this, no clear pathological changes were observed in control and Titan WAT. Titan mice also showed multifocal fatty cells in the pancreas, termed pancreatic lipomatosis or steatosis, without atrophic or inflammatory changes of the adjacent parenchyma, a phenotype not present in control animals (Fig. 1i).

Previous work suggested that obesity can cause alterations in the brain^31,32^. Indeed, hematoxylin and eosin (H&E)-stained sections of the brains of all five 16–17-week-old Titan mice analyzed showed morphological changes in the hippocampal dentate gyrus (Supplementary Fig. 4). This phenotype was not observed in any of the six age-matched control animals studied.

Notably, the lifespan of male Titan mice was dramatically shorter than that of control mice (Fig. 1j). Under standard breeding feed (SBF), Titan mice reached 10 and 50% mortality at 125 and 325 days, respectively. Their average and maximal lifespans were 317.5 and 614 days. In comparison, control mice showed double mean lifespan, reaching 10 and 50% death at 307 and 645 days, respectively. The shortened lifespan of Titan mice combined with obese metrics suggests that the Titan mice suffer from MUO rather than exhibiting MHO.

### Association between phenotypic features of Titan mice and regions of distinct genetic differentiation

Based on the phenotypic analysis, we hypothesized that several genetic alterations in Titan mice would translate to the observed phenotypes (metabolic regulation, weight, and size). We first assessed the genetic differentiation associated with the Titan mouse compared to the unselected FZTDU control line and all the other Dummerstorf long-term selected mouse lines^9^. For this, we used the whole genome sequencing data recently generated from the ‘Dummerstorf mouse lines’^ 9^. One of these Dummerstorf mouse lines is the DU6P line, which originated from the same original mouse colony as the Titan mice and was selected for high protein mass at 6 weeks of life, resulting in a giant but lean phenotype^13^. We first described regions of distinct genetic differentiation (RDD) unique to Titan mice and identified 84 genes (Supplementary Data 1). Since both Titan and DU6P are giant mice (see ref. ^9^), an overlap of RDD was expected between both lines. A second analysis excluding the DU6P mice was conducted and identified 173 protein-coding genes (335 including non-coding genes) in RDD unique to Titan mice (Supplementary Data 2). Gene Ontology (GO) enrichment analysis (Biological Process) of these Titan-specific RDD genes revealed an association with several biological processes, including metabolic regulation (*Kat2a*, *Sfrp5*, *Agpat4*, and *Hcrt*), growth factor signaling (*Stat3*, *Stat5a*, *Stat5b,* and *Igf2r*), skin differentiation and immune regulation (Table 1).

**Table 1:** Gene ontology (GO) term analysis of Titan-specific RDD (regions of distinct genetic differentiation) genes shows enrichment of altered genes in different biological processes connected to growth and metabolic regulation.

GO ID	Term name	Enrichment ratio	FDR	NCBI gene ID	Gene symbol
GO:0031424	Keratinization	21.7	0.020	20754; 20762; 20763; 20765; 20766	Sprr2h; Sprr2i; Sprr2k; Sprr1b; Sprr3
GO:0044262	Cellular carbohydrate metabolic process	5.3	0.050	11677; 12015; 14042; 14534; 15531; 20848; 50798; 50873; 58246; 66646	Rpe; Gne; Slc35b4; Akr1b3; Kat2a; Stat3; Ext1; Prkn; Ndst1; Bad
GO:0060343	Trabecula formation	22.5	0.050	14225; 15214; 21687; 170826	Fkbp1a; Tek; Hey2; Ppargc1b
GO:0060416	Response to growth hormone	21.7	0.050	20848; 20850; 20851; 227231	Cps1; Stat5b; Stat5a; Stat3
GO:0009913	Epidermal cell differentiation	6.1	0.065	15214; 20754; 20762; 20763; 20765; 20766; 22283; 67694	Ush2a; Sprr2h; Sprr2i; Sprr2k; Sprr1b; Sprr3; Ift74; Hey2
GO:0010918	Positive regulation of mitochondrial membrane potential	35.1	0.068	12015; 50873; 269523	Vcp; Prkn; Bad
GO:0072044	Collecting duct development	35.1	0.068	11677; 71228; 240025	Akr1b3; Dlg5; Dact2
GO:0060396	Growth hormone receptor signaling pathway	32.6	0.074	20848; 20850; 20851	Stat5b; Stat5a; Stat3
GO:0060347	Heart trabecula formation	30.4	0.074	14225; 15214; 21687	Fkbp1a; Tek; Hey2
GO:0045579	Positive regulation of B cell differentiation	30.4	0.074	12015; 20850; 20851	Stat5b; Stat5a; Bad
GO:0046629	Gamma-delta T cell activation	28.5	0.075	20850; 20851; 270152	Jaml; Stat5b; Stat5a
GO:0071378	Cellular response to growth hormone stimulus	28.5	0.075	20848; 20850; 20851	Stat5b; Stat5a; Stat3
GO:0050891	Multicellular organismal water homeostasis	14.5	0.075	11677; 20276; 22393; 229574	Flg2; Wfs1; Akr1b3; Scnn1a
GO:0035966	Response to topologically incorrect protein	7.4	0.075	22393; 50873; 56323; 69310; 69601; 269523	Dab2ip; Dnajb5; Vcp; Wfs1; Pacrg; Prkn
GO:0072528	Pyrimidine-containing compound biosynthetic process	14.1	0.075	29807; 68262; 69692; 227231	Cps1; Tpk1; Hddc2; Agpat4
GO:0030216	Keratinocyte differentiation	7	0.086	20754; 20762; 20763; 20765; 20766; 67694	Sprr2h; Sprr2i; Sprr2k; Sprr1b; Sprr3; Ift74
GO:0030104	Water homeostasis	13.2	0.086	11677; 20276; 22393; 229574	Flg2; Wfs1; Akr1b3; Scnn1a
GO:0045838	Positive regulation of membrane potential	24	0.086	12015; 50873; 269523	Vcp; Prkn; Bad
GO:0030856	Regulation of epithelial cell differentiation	6.7	0.093	12015; 15214; 20850; 20851; 21937; 74123	Tnfrsf1a; Hey2; Stat5b; Stat5a; Foxp4; Bad
GO:0061383	Trabecula morphogenesis	12.4	0.094	14225; 15214; 21687; 170826	Fkbp1a; Tek; Hey2; Ppargc1b
GO:0043588	Skin development	4.7	0.095	20754; 20762; 20763; 20765; 20766; 67694; 229574; 240025	Sprr2h; Sprr2i; Sprr2k; Sprr1b; Sprr3; Flg2; Ift74; Dact2

*p* value adjustment with Benjamin–Hochberg procedure for false discovery rate (FDR).

Next, we sought to further explore potential links between genotype and phenotype. Skin thickness has been shown to correlate with body mass index (BMI) positively^33^. As expected, Titan mice displayed a thicker dermis of the skin compared to the control group (Supplementary Fig. 5a). We also found that the inflammatory cytokines interleukin 6 (IL-6) and tumor necrosis factor-alpha (TNFα) were significantly increased in the plasma of Titan mice compared to unselected controls (Supplementary Fig. 5b, c). As a further indication of inflammation, histological analysis of the thymus revealed the presence of nodular thymic medullary hyperplasia in Titan mice (Supplementary Fig. 5d). Immunohistochemistry of these medullar nodes revealed that they were composed mainly of B cells (Supplementary Fig. 5d). Thus, our data support a link between Titan-specific RDD such as metabolic regulation, growth, skin differentiation, and immune regulatory genes with Titan-specific phenotypes of obesity, size, thicker dermis, and increased hallmarks of inflammation.

### Altered histone 4 acetylation levels in male Titan mice

**Fig. 2:**
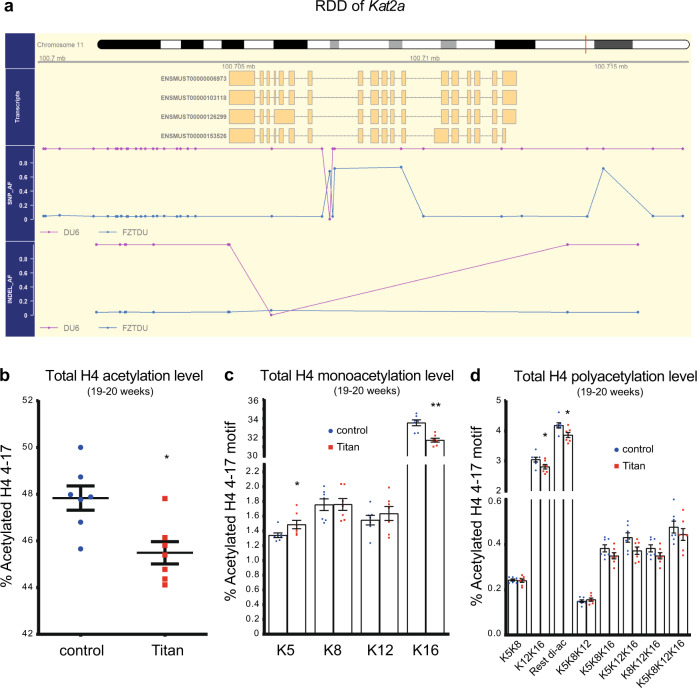
Histone 4 acetylation levels are altered in Titan compared with unselected control mice. **a** Scheme of the regions of distinct genetic differentiation (RDD) of the *Kat2a* gene in Titan and control mice. **b** Total levels of histone 4 (H4) 4–17 levels via mass spectrometry (*p* = 0.0111, MWU-test) (*n* = 7 per group). **c** Quantification of monoacetylation of histone 4 4–17. *p* values, Mann–Whitney *U* (MWU)-test (H4K5K8) = 0.0175, (H4K8) = 0.901, (H4K12) = 0.455, (H4K16) = 0.0023 (*n* = 7 per group). **d** Quantification of polyacetylation of histone 4 4–17. *p* values, MWU-test (H4K5K8) = 1, (H4K12K16) = 0.0379, (H4-rest di-ac) = 0.0379, (H4K5K8K12) = 0.62, (H4K5K8K16) = 0.208, (H4K5K12K16) = 0.053, (H4K8K12K16) = 0.208, (H4 tetra-ac) = 0.62 (*n* = 7 per group). *p < 0.05, **p < 0.01. Error bars indicate SEM.

Genetics and epigenetics functions are intimately linked, and their alterations are associated with disease^34^. We identified *Kat2a* (*Gcn5*) as one of the genes associated with specific genetic differentiation in Titan mice (Fig. 2a and Supplementary Data 1; Allele frequency = 0.97). *Kat2a* encodes a histone acetyltransferase, which has also been implicated in metabolic and energy regulation^35^, thus raising the possibility that histone acetylation might be altered in Titan mice. To address this suggestion, we compared histone 4 acetylation levels of 10-week-old and 19–20-week-old Titan vs. control mice by mass spectrometry^36,37^. For this analysis, we focused on the liver, of central importance for fat and carbohydrate metabolism, and KAT2A regulates metabolic activity in this organ^38^.

At 19–20 weeks of age, we observed significantly lower levels of histone 4 acetylation in Titan vs. control mice (Fig. 2b). Highly abundant histone 4 acetylation motifs in mammals such as H4K16 and H4K12K16^37^, as well as poly-acetylated motifs were significantly reduced in Titan vs. control mice (Fig. 2c, d). By contrast, the H4K5 acetylation motif, which was shown to be moderately regulated at least in part by KAT2A^36^, was significantly higher in Titan mice compared to control mice (Fig. 2c). Of note, comparable results were obtained in younger Titan mice (10 weeks of age, see deposited data sets). Taken together, Titan mice display considerable alterations in various histone acetylation motifs compared to the unselected mouse line which might manifest in altered transcriptomes^39^.

### Male hybrids of Titan and control mice show intermediate phenotypes

**Fig. 3:**
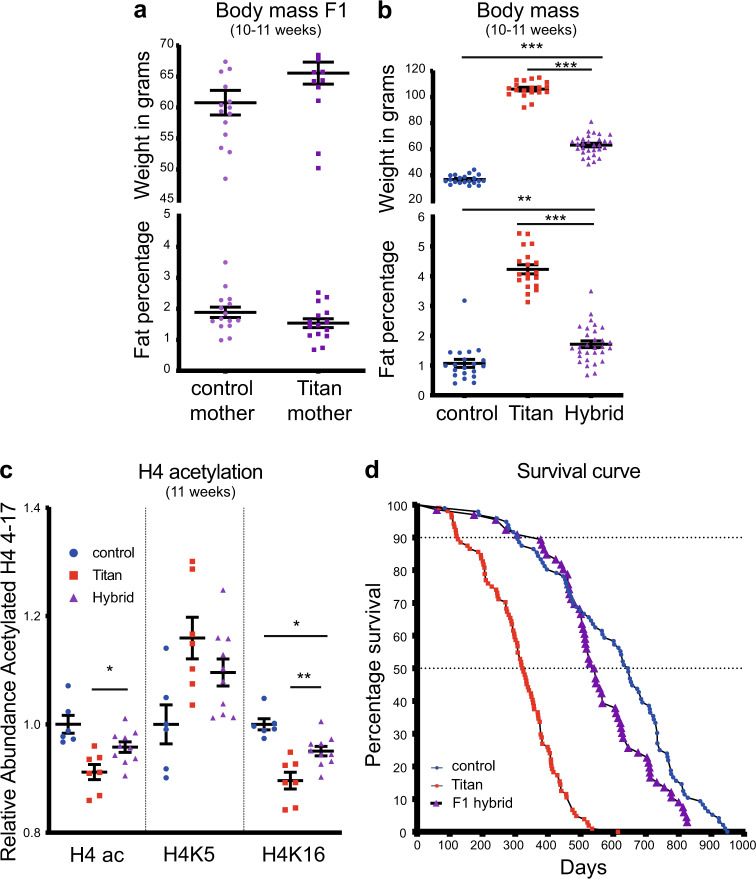
Hybrid offspring of control and Titan mice display intermediate obesity and lifespan. **a** Body weight and visceral fat percentage in F1 hybrids (offspring of mother control/Titan x father Titan/control, respectively; *n* = 15 each), Mann–Whitney *U* (MWU)-test was used to calculate *p* value. **b** Body weight and visceral fat percentage in control (*n* = 20), Titan (*n* = 19), and hybrid (*n* = 30). One-way ANOVA followed by Tukey test *p* value: control vs. hybrid (weight) <0.0001, (%visceral fat) = 0.0021 and Titan vs. hybrid (weight) <0.0001 (%visceral fat) <0.0001. **c** Quantification of total histone 4 (H4) 4–17 acetylation, H4K5ac and H4K16ac in control (*n* = 6), Titan (*n* = 7), and hybrid (*n* = 10) mice. One-way ANOVA followed by Tukey test *p* value: control vs. hybrid (H4ac) = 0.0765 (H4K5) = 0.116, (H4K16) = 0.0184 and Titan vs. hybrid (H4ac) = 0.0383, (H4K5) = 0.330, (H4K16) = 0.0065. **d** Comparison of lifespans and mortality rates of Titan and control male mice to hybrid (*n* = 66). *p < 0.05, **p < 0.01. ***p < 0.001. Error bars indicate SEM.

We next assessed the potential contribution of Titan mice's maternal impact and phenotypic features. In order to address this, we generated hybrids by crossing Titan and unselected control mice (mother control/Titan x father Titan/control, respectively). Male F1 hybrids of 10–11 weeks of age showed similar weight and fat percentage independently of the genotype of the mother (Fig. 3a).

Notably, the combined population of male F1 hybrids showed intermediate body weight and fat percentage compared to control and Titan mice at the same age (Fig. 3b). Similarly, the hybrid mice showed intermediate histone 4 acetylation levels in the liver, including at H4K5 and H4K16 (Fig. 3c). Furthermore, the lifespan of hybrid mice, although improved compared to Titan mice, was reduced compared to that of control mice (Hybrid vs. Titan log-rank test; *p* < 0.0001, *χ*^2^ = 93.2 Control vs. hybrid, log-rank test; *p* = 0.0552, *χ*^2^ = 3.676). The median lifespan of hybrids was 543.5 days vs. 325 (Titan) and 645.5 days (control) (Fig. 3d). These observations illustrate the quantitative genetic background determining mice obesity and lifespan.

### Male Titan mice display altered liver metabolism along with changes in gene and protein expression

**Fig. 4:**
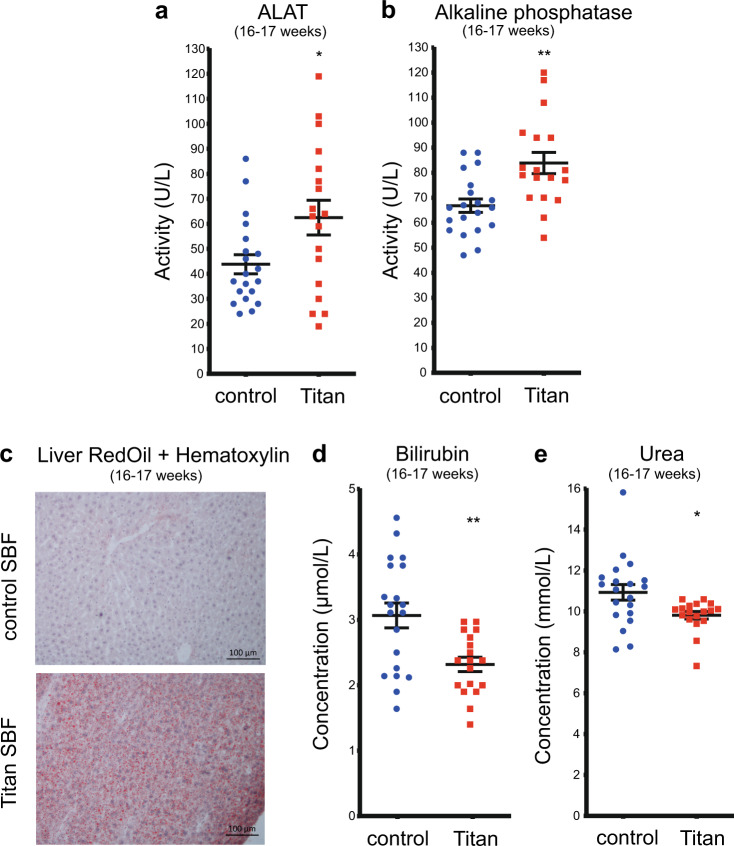
Titan mice display altered liver metabolism. **a** ALAT (*p* = 0.0262) and (**b**) ALP (*p* = 0.0019) levels in control (*n* = 20) and Titan mice (*n* = 18). **c** Oil Red O with hematoxylin staining of fat in liver of control and Titan mice (*n* = 4 per group). **d** Bilirubin levels in control (*n* = 20) and Titan mice (*n* = 18; *p* = 0.0019). **e** Urea levels of control (*n* = 20) and Titan (*n* = 18) mice (*p*  = 0.0132). *p < 0.05, **p < 0.01. Unpaired two-tailed *t-*tests with Welch’s correction were used to calculate *p* values. Error bars indicate SEM.

We next evaluated possible alterations in liver function and metabolism in male Titan mice. First, alanine aminotransferase (ALAT) and alkaline phosphatase (ALP) activity, markers of potential liver damage^40^, were significantly elevated in Titan mice at 16–17 weeks of age (Fig. 4a, b). Combined Oil Red O and hematoxylin staining of the liver also revealed numerous small fat droplets in Titan mice (Fig. 4c). However, no clear signs of hepatic steatosis were found by H&E staining of 16–17-week-old Titan liver tissue (Supplementary Fig. 6). Bilirubin, a waste product of heme metabolism in the liver, was significantly lower in Titan mice than in controls (Fig. 4d). In addition, plasma urea levels were also decreased in Titan mice (Fig. 4e), pointing to the dysregulation of the urea cycle and protein metabolism in the liver. Altogether, these findings suggest an altered liver metabolism in Titan compared to control unselected mice.

**Fig. 5:**
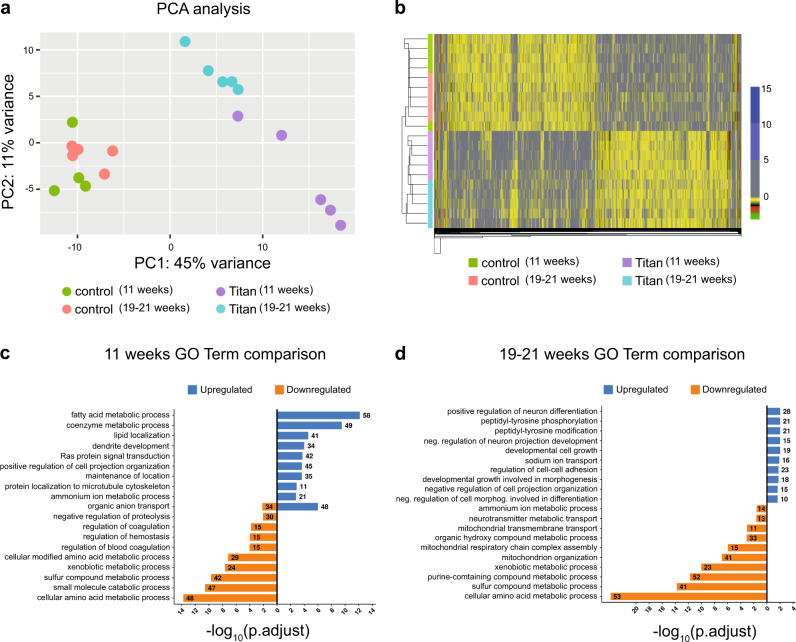
Liver transcriptome analyses reveal differential expression of genes associated with metabolic pathways in Titan mice. **a** Principal component analysis (PCA) of liver transcriptomes revealed clustering differences between Titan mice and controls. Control mice at 11 weeks and 19–21 weeks had similar transcriptomes, while younger and older Titan mice had distinct transcriptomes. **b** Heatmap showing significantly altered genes in control and Titan mice at 11 weeks and 19–21 weeks of age. The color gradient in each cell in the heatmap represents the scaled normalized log counts from each replicate and condition. **c** Gene ontology (GO) term analysis in 11-week-old Titan mice compared with corresponding controls. **d** GO terms in 19–21-week-old Titan mice compared with the corresponding controls (*n* = 5 per group).

We hypothesized that Titan mice, unlike control animals, would display a transcriptome profile that is linked to increased body fat and altered metabolism. To identify pathways regulated at the transcriptional level in the liver, we performed RNA-sequencing (RNAseq) on this organ from 11-week-old and 19–21-week-old mice. Principal component analysis (PCA) revealed distinct Titan and control transcriptomes (Fig. 5a). Gene expression in younger and older controls clustered together, but in Titan mice, the expression profile shifted between the age groups (Fig. 5a), indicating age-dependent effects in Titan but not in control mice.

Compared to age-matched controls, 11-week-old Titan mice exhibited 1150 upregulated and 1227 downregulated genes (Fig. 5b, Supplementary Fig. 7a, and Supplementary Data 3,4) and 19–21-week-old Titan mice 693 upregulated and 828 downregulated genes (Fig. 5b and Supplementary Fig. 7d). We observed a significant overlap of transcriptomic alterations between both age group comparisons, with 402 upregulated and 461 downregulated genes.

We next compared 11-week-old Titan to control mice. The top ten upregulated GO terms highlighted genes involved in several central metabolic processes (Fig. 5c). Central metabolic regulators such as *mTOR, Elovl5, Elovl6*, and acetyl-CoA carboxylase were upregulated (Supplementary Data 3, 4), suggesting altered fat metabolism in the liver. The top ten downregulated GO terms included xenobiotic, amino acid, and sulfur metabolism (Fig. 5c). For example, many cytochrome P450 family genes, including *Cyp2c37*, were downregulated in 11-week-old Titan mice compared to controls at the same age (Supplementary Data 3, 4). Interestingly, cytochrome P450 family genes are downregulated in a high-fat diet^24^. Furthermore, several genes related to methionine, folate cycle, and hydrogen sulfide production (e.g., *Bhmt, Gnmt, Cth* (*Cgl*)*, Dmgdh*), as well as *Hcrtr2* were downregulated in 11-week-old Titan mice.

The top ten upregulated GO terms in 19–21-week-old Titan mice compared with control revealed the upregulation of genes involved in neuronal differentiation and peptidyl-tyrosine phosphorylation activity (Fig. 5d). The top ten downregulated GO terms in 19–21-week-old Titan mice revealed genes involved in regulating xenobiotic, amino acid, and sulfur metabolism (Fig. 5d), which is similar to the comparison at 11 weeks of age (Fig. 5c). Of note, genes involved in the mitochondrial organization and mitochondrial respiratory complex assembly were downregulated as well (Fig. 5d). We found that *Acss2*, an acetyl-CoA synthesis enzyme linked with histone acetylation^41^, was downregulated in Titan mice.

Compared to 11-week-old Titan mice, 19–21-week-old Titan mice had increased expression of several enzymes involved in glucose and insulin response (Supplementary Fig. 7b). Also, *Cyp7b1*, which encodes an enzyme involved in cholesterol catabolism converting cholesterol to bile acids and is positively regulated by a high-fat diet^24^, was upregulated in 19–21-week-old compared to 11-week-old Titan mice. On the other hand, the top ten downregulated GO terms highlighted genes involved in fatty acid, coenzyme, and carbohydrate metabolism (Supplementary Fig. 7c). Taken together, liver transcriptomic analysis demonstrates several metabolic alterations in Titan mice.

Next, we compared the liver proteomes of Titan and control mice (Supplementary Data 5). As with the RNAseq data, larger proteomic changes were seen in the 11-week-old Titan to control comparison than in the 19–21-week-old comparison (Supplementary Fig. 8a, b). Relative to age-matched controls, 11-week-old Titan mice exhibited 207 upregulated and 289 downregulated proteins, while 19–21-week-old Titan mice showed 125 upregulated and 142 downregulated proteins (Supplementary Fig. 8a, b). Consistent with the transcriptome results, pathway analysis revealed increased fatty acid and coenzyme metabolism, notably pathways involved in fat utilization (ACACA, IDH1, and ACOX1) and in lipid biosynthesis (FASN and ACSL5). In contrast, downregulation in xenobiotic, amino acid, and sulfur metabolism was observed in 11-week-old Titan mice (Supplementary Fig. 8c).

Proteins involved in lipid metabolism were also enriched in 19–21-week-old Titan mice compared to controls (Supplementary Fig. 8d), although this was not observed in the RNAseq top ten GO terms (Fig. 5d). We also found that proteins upregulated in 19–21-week-old Titan mice were associated with carbohydrate catabolism and coenzyme metabolism (Supplementary Fig. 8d), while downregulated proteins were enriched in amino acid and sulfur metabolism (Supplementary Fig. 8d), in line with the RNAseq analysis (Fig. 5d). As with the transcriptomic data, we observed a reduction in the abundance of BHMT, GNMT, and CTH (CGL).

Collectively, both transcriptomics and proteomics data hint toward increased fat metabolism in Titan compared to control mice.

### Late dietary intervention at 12 weeks of age improves the obesity and lifespan phenotypes in Titan mice

We next investigated the impact of dietary intervention on measures of obesity, epigenetic markers, liver gene expression patterns, and lifespan of Titan mice. We compared the impact of a diet with moderate energy reduction using energy-reduced feed, hereafter referred to as ERF (see Methods), to that of SBF. We introduced ERF at 12 weeks of age onward and termed this diet intervention as late intervention due to the fact that 10 and 25% of the Titan population were already dead by 18 and 33 weeks, respectively.

**Fig. 6:**
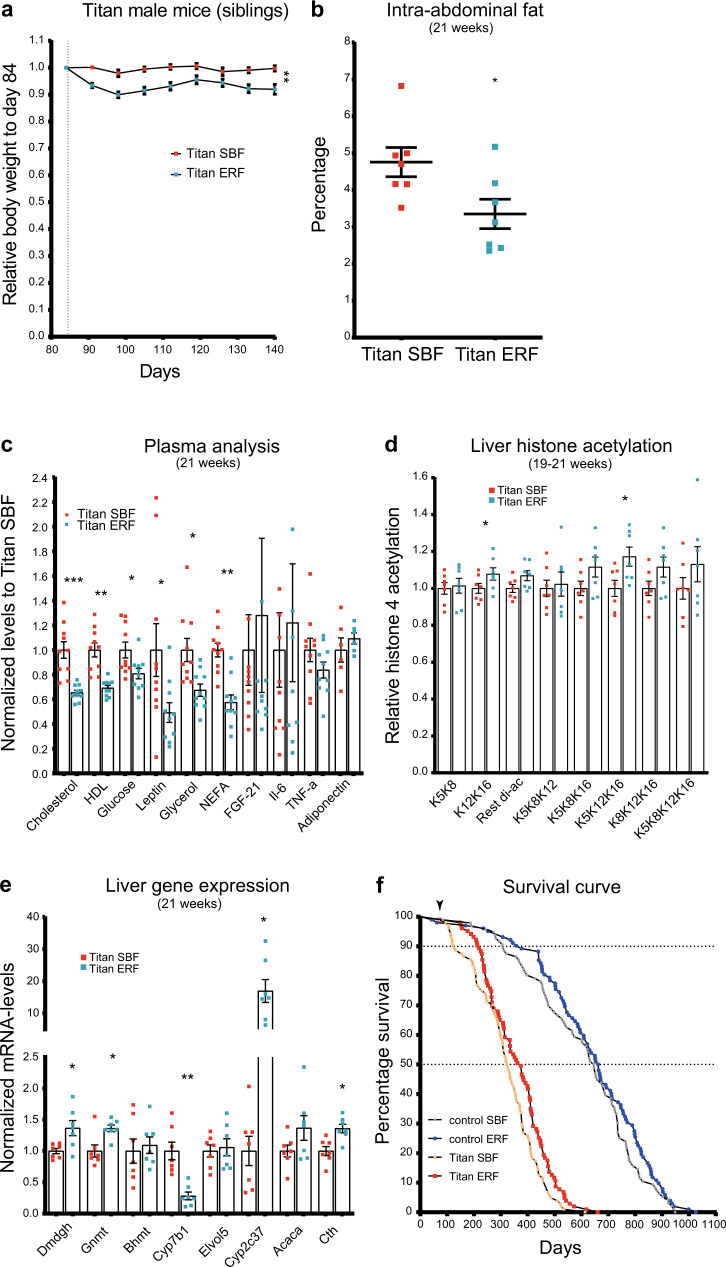
Late dietary intervention by switching to ERF improves health metrics and extends the lifespan of Titan mice. **a** Switching standard breeding feed (SBF) to energy-reduced feed (ERF) at 12 weeks resulted in a persistent average weight loss in Titan siblings (*n* = 23 per group). Mann–Whitney-*U* (MWU)-test was calculated for day 140, *p* = 0.0011. **b** Compared to age-matched control Titan mice, ERF-fed Titan mice had a lower percentage of intra-abdominal fat at 21 weeks (*n* = 7 per group). Paired Wilcoxon-test, *p* = 0.0313. **c** Late ERF feeding at 12 weeks of age decreased the levels of plasma cholesterol (pval.adj = 0.00013), high-density lipoprotein (HDL) (pval.adj = 0.0013), glucose (pval.adj = 0.043), leptin (pval.adj = 0.043), glycerol (pval.adj = 0.0165), and non-esterified fatty acids (NEFA) (pval.adj = 0.00132) in 21-week-old mice (*n* = 10 per group). MWU-test, by multiple correction to calculate *p* values. **d** Quantification of polyacetylation of histone 4 4–17 following ERF feeding. *p* values, paired MWU-test (H4K5K8) = 0.8125, (H4K12K16) = 0.0469 (H4-rest di-ac) = 0.1563, (H4K5K8K12) = 0.9375, (H4K5K8K16) = 0.0781, (H4K5K12K16) = 0.0313, (H4K8K12K16) = 0.0781, (H4 tetra-ac) = 0.375 (*n* = 7 per group). **e** RT-PCR comparing gene expression of candidate genes from ERF- and SBF-fed Titan mice siblings at 21 weeks of age (*n* = 7 per group). Paired *t-*test was performed followed by multiple correction to calculate adjusted *p* values; pval.adj (*Dmdg*) = 0.032, (*Gnmt*) = 0.012, (*Bhmt*) = 0.736, (*Cyp7b1*) = 0.008, (*Elovl5*) = 0.736, (*Cyp2c37*) = 0.012, (*Acaca*) = 0.0587, (*Cth*) = 0.012. **f** Switching to ERF feeding at 12 weeks of age (vertical black arrowhead) increased the lifespan of both Titan and control mice (Log-rank test; *p* = 0.0087, *χ*^2^ = 6.892 Titan and *p* = 0.162, *χ*^2^ = 1.955 control). The ages at 25, 75, and 90% death and the median lifespan are presented in Supplementary Table 1. *p < 0.05, **p < 0.01, ***p < 0.001. Error bars indicate SEM.

ERF intervention resulted in a 5–10% weight reduction in Titan mice, compared to SBF-fed siblings (Fig. 6a; Mann–Whitney *U* (MWU) test, *p* = 0.0017). By 21 weeks, ERF-fed Titan mice had significantly reduced abdominal fat percentage compared to SBF-fed mice (Fig. 6b; MWU-test, *p* = 0.0313). Also, the ERF regime lowered the levels of plasma cholesterol, HDL, and glucose in Titan mice (Fig. 6c and Supplementary Data 6). Furthermore, plasma levels of leptin, glycerol and non-esterified fatty acids (NEFA) were significantly decreased under the ERF regime (Fig. 6c and Supplementary Data 6). However, the plasma levels of IL-6, TNFα, FGF21, and adiponectin were not significantly changed upon ERF (Fig. 6c).

Late ERF intervention in Titan mice also resulted in a general increase in poly-acetylated motifs of histone 4 levels, which resembled those observed in control mice (Fig. 6d). In general, motifs containing K12K16 acetylation motifs (significant H4K12K16 and H4K5K12K16) were increased in ERF-fed compared to SBF-fed Titan mice (Fig. 6d). In addition, late ERF intervention reversed the expression profiles of some of the investigated genes (Fig. 5 and Supplementary Fig. 7, and Supplementary Data 3). In fact, genes that were downregulated in Titan versus control mice (e.g., *Dmdgh*, *Gnmt*, *Cth*, and *Cyp2c37*) were upregulated in ERF-fed vs. SBF-fed Titan mice (Fig. 6e)^24^. Similarly, upregulated genes in Titan SBF versus control SBF mice (e.g., *Cyp7b1*) were downregulated in ERF-fed vs. SBF-fed Titan mice (Fig. 6e).

Finally, we investigated the impact of the ERF diet on the lifespan of Titan and control mice. The ERF diet significantly increased the lifespan of Titan mice (log-rank test; for *p* = 0.0087, *χ*^2^ = 6.892 Titan mice and *p* = 0.162, *χ*^2^ = 1.955 for control mice) (Fig. 6f). ERF-fed Titan mice reached 10 and 50% death at 219 and 374 days, respectively, which is significantly longer than for the SBF-fed Titan mice (10% death at 125 days and 50% death at 325 days). In comparison, ERF-fed control mice reached 10% death at 377 days vs. 307 days for SBF-fed control mice. The average lifespan of ERF-fed Titan mice was 359.5 days, compared to 317.4 days for SBF-fed Titan mice (Supplementary Table 1).

Taken together, our results indicate that a late ERF intervention could, at least in part, revert the phenotypes of Titan mice associated with MUO and lifespan.

### Female mice display physical features of obesity and increased size

We have also evaluated several phenotypic features of female Titan mice. Titan females weigh significantly more than female control mice (Supplementary Fig. 9a), display a higher percentage of intra-abdominal fat (Supplementary Fig. 9b) and increased plasma leptin levels (Supplementary Fig. 9c), but without alterations in adiponectin (Supplementary Fig. 9d). Surprisingly, we observe no changes in most mRNA levels of selected genes that were altered in Titan males (Supplementary Fig. 9e). Nonetheless, the ERF regime caused a significant weight loss (Supplementary Fig. 9f) but a non-significant decrease in fat percentage (Supplementary Fig. 9g) or changes in leptin and adiponectin levels (Supplementary Fig. 9h). Lastly, in contrast to males, ERF caused no significant alterations in the levels of selected genes in females Titan mice (Supplementary Fig. 9i). Further detailed work is needed to better characterize the female phenotype.

### Late dietary intervention at 12 weeks of age alters the gut microbiome of male and female Titan mice

As diet has a role in determining the composition of the gut microbiome^42^, we characterized the impact of ERF diet on the distribution of the microbiome in cecum digesta of male and female Titan. On the genus level, we found that 14 (males) and 18 (females) microbial taxa were significantly altered in ERF vs. SBF Titan mice. Notably on the genus levels, ten taxa were altered in a similar fashion in males and females. For example, the relative abundance of *Lactobacillus* and *Lachnospiraceae* UCG-001/ge was substantially reduced, while *Candidatus Soleaferrea* and genera of the *Eggerthellaceae* were increased upon ERF diet (Supplementary Fig. 10 and Supplementary Data 7).

## Discussion

In this study, we provide several links between Titan mice’s genotype and their unique phenotypes. GO enrichment of RDD highlighted genetic variability associated with genes involved in multiple pathways including metabolic regulation and growth control. Similarly, RDD genes involved in skin differentiation correlated with our observation that Titan mice display a thicker dermis than control mice, which is in line with previous data linking increased BMI with thicker skin^33^. Several RDD genes linked to immune regulation and inflammation (notably *Stat3* and *Stat5* family members) were also identified^43^, in correlation with Titan mice's immune and inflammatory phenotype. For example, Titan mice show signs of systemic inflammation, such as high plasma levels of IL-6 and TNFα coupled with thymic medullary hyperplasia. Besides being associated with the initiation and progression of multiple diseases^44,45^, elevated circulating IL-6 and TNFα were documented in obese mice and humans^46,47^. Supporting this finding, several mouse models of obesity revealed increased accumulation of macrophages in the liver^48,49^ or in adipose tissue^50^. Increased tissue inflammation in Titan mice might contribute to tissue damage^40^. Notably, one identified RDD contained the *Hcrt* gene, which is a hypothalamic neuropeptide that can regulate feeding behavior and metabolic homeostasis^51,52^. While we did not evaluate gene expression levels in the brain area that may account for metabolic alterations in the Titan mice, we observed a significant reduction of *Hcrtr2* in the liver.

*Kat2a*, which encodes the histone acetyltransferase GCN5, was also part of an RDD specific to Titan mice and is involved in metabolic regulation^35^. An increase in H4K5 acetylation in the liver of Titan mice suggests a possible causal link between the two phenotypes^36^. However, we did not detect significant changes in the expression level of *Kat2a* in the liver of Titan mice. Although it is unlikely that changes in H4K5 acetylation are the consequence of changes in *Kat2a* expression, we cannot exclude that they are related to changes in *Kat2a* enzymatic activity or to alterations in other GCN5-related genes. Additional investigations will be necessary to address this question and further assess alterations in expression and activity of histone acetyltransferases in Titan mice, in correlation with changes in histone acetylation levels.

Notably, F1 hybrids show intermediate body weight and fat percentage, histone 4 acetylation levels, and lifespan. The intermediate phenotype appears to be independent of the mother’s genotype (control or Titan), thus demonstrating a weaker link between maternal inheritance and weight or fat levels. Further analyses of F2 hybrids are required to link specific genes to the particular phenotypes of the Titan mice described in this study^4,53^.

Previous research at earlier generations suggested that Titan mice display benign obesity (MHO) without additional comorbidities^5^. In contrast, we now show that current generations of the Titan mice indeed exhibit detrimental obesity, including several hallmarks that are attributed to MetS, such as increased fat deposition in tissues, high plasma levels of fasting triglycerides, fasting cholesterol, FGF21^19,20,54^, and insulin^16,55^, as well as early signs of heart fibrosis^21^. Titan mice also showed whitening of BAT, and ectopic fat in the pancreas, both linked to obesity and MetS^22,56^. Increased pancreatic fat might have implications for the onset of type 2 diabetes^57^, which is frequently associated with insulin resistance. Importantly, obesity is known to be associated with reduced life expectancy and various conditions related to aging, such as inflammation^58–63^. Indeed, we found that Titan mice exhibited a shorter lifespan compared to control mice, which is in line with a recent study showing that female Titan mice show a reduced lifetime fecundity compared to the unselected control mice^64^.

Alterations in hepatic function were evidenced by epigenomic, transcriptomic, and proteomic changes detected in the liver of Titan mice. Genes and proteins involved in lipid metabolism and biosynthesis were consistently upregulated in Titan compared to control mice, which might account for the high plasma levels of cholesterol and fasting triglycerides observed in this model. On the other hand, compared to controls, Titan mice exhibited several downregulated genes and proteins associated such as cytochrome P450. These downregulations might indicate altered endogenous metabolite metabolism^65^, increased liver inflammation^66,67^, and/or a generally reduced capacity to deal with toxic compounds in the liver. Xenobiotic metabolism strongly impacts the ability of the organism to maintain homeostasis and cope with disease^68^, which may contribute to the increased morbidity of these mice. Also, Titan mice showed a reduced expression of genes related to the folate cycle and its downstream effectors, such as *Ahcy*, *Gsta* family, *Gnmt*, and *Cth*. Such genes were also shown to be downregulated in the mouse liver upon a high-fat diet^24^. Similarly, genes involved in mitochondrial organization and function were downregulated in 19–21-week-old Titan mice, which may hint toward altered mitochondrial activity in older Titan mice^61^. Altogether, these findings suggest an altered liver metabolism in Titan compared to control mice, along with their obese phenotype and shorter lifespan.

Interestingly, the liver of Titan mice showed overall decreased histone 4 acetylation at the 4–17 motif, specifically histone acetylation motifs such as H4K16 and H4K12K16 acetylation, which is in correlation with a reduction in the expression of acetyl-CoA synthases (*Accs2* and *Pdhx*) which may imply reduced acetyl-CoA availability. This could underlie the general alteration in histone acetylation levels^41,69^ and might also link this epigenetics phenotype to lifespan regulation^70,71^.

Our data support the notion that Titan mice are a potential translational model, as ERF feeding introduced at 12 weeks of age could partially reverse the phenotypes associated with obesity, lifespan, and histone 4 acetylation patterns. Notably, intra-abdominal fat was reduced at 21 weeks and plasma levels of leptin, glycerol, and NEFA, which are linked to obesity^16,17,72,73^, were decreased in ERF-fed male Titan mice. This phenotype improvement is reminiscent of other time-restricted fasting animal models or patients with MetS^74^. ERF feeding seems to specifically increase levels of the poly-acetylated histone 4 motif, including K12K16 acetylation. These modifications might impact transcriptional regulation important for mediating the benefits of a healthy diet in obese animals and maybe novel therapeutic targets to treat obesity. In line with these data, we also found that ERF feeding partially reversed the expression pattern of key metabolic enzymes (e.g., *Dmdgh, Gnmt, Cth,* and *Cyp2c37*) in the liver^24^. Several of these genes were implicated in mediating the benefits of caloric restriction^75–77^. For example, CTH (CGL) affects the dietary restriction response in mice via H_2_S production^78^. Similarly, feed intake restriction increases GNMT levels, promotes energy homeostasis, and increases a healthy lifespan^77,79^. Of note, the ERF feeding caused alterations in the abundance of various microbial genera that have been implicated in diet and health. For example, ERF decreases several *Lachnospiraceae* genus while a recent study showed that intestinal colonization of a *Lachnospiraceae* strain in ob/ob mice caused an increase in fasting plasma glucose, adipose tissue, and overall weight^80^. Altogether, our data demonstrate that Titan mice are responsive to diet intervention, and thus may represent a suitable translational mouse model to assess the impact of diet, microbiome, or other therapeutic interventions on manifestations of obesity^16,29,81^.

While our study focused on characterizing male Titan mice, we also provide a basic characterization of Titan females. Although female Titan mice are large, obese, and display high levels of leptin, our initial data suggest molecular sexual dimorphism. We conclude that more work is needed, e.g., including survival assessment, organ histology, or transcriptome analysis, to thoroughly characterize the females.

The polygenetic Titan mouse model may present several advantages over other currently used mouse models of unhealthy obesity. Notably, no further genetic alterations or elimination of regulatory genes are needed to achieve the obesity phenotype in Titan mice. Most genetic rodent models of obesity are based on the disruption of the leptin signaling pathway. This is true for both mice (leptin-deficient ob/ob, leptin receptor-deficient db/db, or POUND C57BL/6NCrl-Lepr db-lb/Crl) and rats (Zucker Diabetic Fatty, DahlS.Z-Lepr^fa^/Lepr^fa^ -DS/obese- and Koletsky rats carrying a nonsense mutation in the leptin receptor)^16,81,82^. Interestingly, other rodent obesity models, such as the UCD-T2DM rat harboring pancreatic defects and the inbred polygenic New Zealand mouse^83^, possess intact leptin signaling. A major disadvantage of some of these rodent models is that they do not closely reflect human obesity, which is multifactorial and not always affecting leptin signaling, thus rendering it difficult to translate findings in these rodents to human patients. Another advantage of the Titan mice, notably when studying the impact of obesity on the lifespan phenotype, is that ERF diet intervention could be already assessed in 4–5 months with Titan mice, whereas a similar intervention would take much longer in other mouse models^29^

**Fig. 7:**
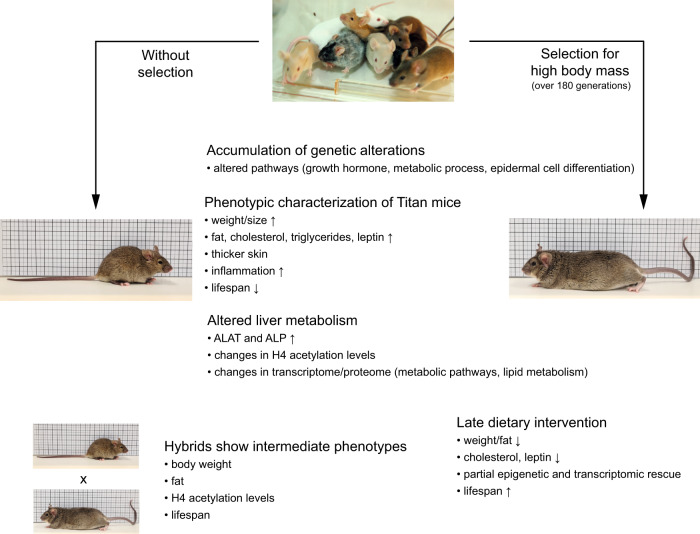
Accumulation of genetic, epigenetic, and physiological alterations during the selection process of Titan mice. Working model of the selection process for body mass over 180 generations in Titan animals. The long selection process resulted in the accumulation of genetic and epigenetic alterations that caused a shift in the transcriptome and proteome, ultimately leading to alterations in metabolic processes such as lipid metabolism. The selection resulted in morphological changes in various tissues such as fat, pancreas, thymus, heart, brain, and skin. These overall changes resulted in giant and extremely obese mice, while unselected mice stemming retained similar characteristics compared to the original mouse colony.

In conclusion, the Titan mouse line represents a significant scientific achievement in breeding of mice and is one of the biggest laboratory mouse lines ever described. The detailed genomic, epigenetic, and phenotypic analyses described here strongly support the Titan line as a relevant model to study various aspects of interventions in unhealthy obesity. We propose a working model which integrates our data and illustrates the various alterations that accompanied the selection of the Titan mice (Fig. 7). Thus, the present phenotypic analysis provides a solid basis for further characterization of the Titan mouse model as a novel tool for studying and potentially developing pharmaceutical interventions targeting obesity and associated disorders.

## Methods

### Animals and housing conditions

All procedures were performed in accordance with national and international guidelines and ethically approved by our own institutional board (Animal Protection Board from the Leibniz Institute for Farm Animal Biology (FBN)). At the German Mouse Clinic (GMC), mice were maintained in individually ventilated cages with water and standard mouse chow according to the directive 2010/63/EU, German laws, and GMC housing conditions (www.mouseclinic.de). All tests were approved by the responsible authority of the district government of Upper Bavaria, Germany.

At the FBN, the animals were maintained in a specific pathogen-free (SPF) environment with defined hygienic conditions at a temperature of 22.5 ± 0.2 °C, at least 40% humidity and a controlled light regime with a 12:12 h light-dark cycle. The mice were kept in polysulfone-cages of 365 × 207 × 140 mm (H-Temp PSU, Type II L, Eurostandard, Tecniplast, Germany) and had free access to pellet feed and water. Mice were fed ad libitum using a SBF (gross energy (GE) = 16.7 MJ kg^−1^, metabolizable energy (ME) = 14.0 MJ kg^−1^, Supplementary Table 2) including 12% fat, 27% protein, and 61% carbohydrates (ssniff^®^ M-Z autoclavable, Soest, V1124-300, Germany).

For the energy-reduced survival experiment, the mice were fed with a mouse maintenance ERF (GE = 15.7 MJ kg^−1^, ME = 11.7 MJ kg^−1^, Supplementary Table 2) characterized by a low energy density and high fiber content including 10% fat, 21% protein, and 69% carbohydrates (ssniff^®^ M-H autoclavable, Soest, V1574-300, Germany). Mice were placed under the ERF diet starting at 12 weeks of age.

In both control and Titan lines, the litter size was standardized to 10 (5 male/5 female pups) immediately after birth until weaning at the age of 21 days. At this age, males of different litters were grouped in a cage with three animals per cage. The first survival experiment was done with *n* = 104 Titan males from generation 181 and *n* = 96 control males originating from generation 192. The second survival experiment under the ERF diet started with *n* = 103 Titan and *n* = 102 control males, one generation later. Additionally, 27 Titan male full sibling pairs of generation 183 were divided into two contemporaneous groups and were fed with SBF or ERF after 12 weeks of age to generate the samples to analyze metabolic parameters. Individual weights of all animals were taken regularly every three weeks, starting with the age of 21 days. Hybrid mice were generated by crossing unselected control mice to Titan mice (Titan mother × control father or control mother × Titan father).

During the survival experiments, all included mice were observed daily for their health condition. If physical impairments were detected, which would cause considerable suffering or substantial pain to the animals, they were sacrificed, and such incidents were documented accordingly.

### Origin of the growth-selected and the control strains

We used mice of an unselected strain (FZTDU) as control and a strain selected for high body mass at day 42 of age (DU6/Titan), both bred at the Leibniz Institute of Farm Animal Biology (FBN), Dummerstorf, Germany.

The initial population of mice (FZTDU) was created in 1969/1970 by crossing four outbred (NMRI orig., Han:NMRI, Han:CFW, Han:CF1) and four inbreds (CBA/Bln, AB/Bln, C57BL/Bln, and XVII/Bln) populations^7^. Mice of the control line FZTDU used in this experiment were mated randomly over about 192 generations with a population size of 100 to 200 mating pairs per generation, respectively. Four generations of the control line are generated yearly using a rotation procedure of Poiley (1960) to avoid inbreeding^84^.

The growth selection started in 1975, thus creating the Dummerstorf growth line DU6 (Titan) by selecting for high body mass by sib selection in a conventional environment. In every generation, four generations per year, 60–80 mating pairs were mated at the age of 63 ± 3 days^8,9^. The selection procedure as described above was maintained for 153 generations. Only seven males and seven females of the DU6 line belonging to generations 154 and 155 (the year 2011–2012), respectively, were used as new founder animals after an embryo transfer in a newly built animal facility. Of note, in 2011/2012, the Titan mouse lines (generations 154 and 155) were transferred into a new state-of-the-art pathogen-free animal facility and their diet composition changed at that point^12^. Over the entire term of the following five generations, the new breeding population of at least 60 pairs of Titan DU6 mice was established^9^, taking care of an equal distribution of the alleles of the 14 founder animals. In opposition to the former sib selection in generation number 161, breeding value estimation started introducing a two-trait BLUP (Best Linear Unbiased Prediction) animal model for male and female body mass. The raising inbreeding coefficient in the selection line was then controlled by the “Optimal Genetic Contributions to the Next Generations” method of Meuwissen^85^.

### German mouse clinic

Importantly, because Titan mice are not inbred, we increased the number of animals from 15 (standard GMC animal number per group and sex for inbred mice) to 20, using only males.

Two cohorts of 20 Titan and 20 control FZTDU male mice were subjected to an extensive phenotypic screening at the GMC, including standardized phenotyping, e.g., in the areas of energy metabolism, clinical chemistry, and pathology^86^ (see also www.mouseclinic.de). Measurements were taken from weeks 11 to 21. The phenotypic tests were part of the GMC screening and performed according to the standardized protocol as described before^87–89^. Variations in protocols are specified.

Animal numbers varied depending on the test performed, as indicated in the respective figure or table.

Plasma clinical chemistry analyses included determining blood lipid and glucose levels on freshly collected Li-heparin-plasma samples collected after overnight feed withdrawal and measuring a broad spectrum of parameters including ALAT and ALP activity, urea, and bilirubin levels in samples collected in ad libitum fed state during the final blood withdrawal. Analyses were performed using an AU480 clinical chemistry analyzer (Beckman-Coulter, Germany). Samples collected during final blood withdrawal were frozen and stored at −80 °C until the day of analysis.

The IL-6, TNFα, Insulin, leptin, and FGF21 plasma levels were determined with a combined electrochemiluminescence multiplexed assay system (Meso Scale Discovery, MSD, Rockville, MD USA). Serum adiponectin and leptin were also measured using standard ELISA according to the manufacturer’s instructions (mouse Adiponectin ELISA, MRP300, R&D Systems; mouse Leptin ELISA, #900300, Crystal Chem).

X-ray imaging was performed in an UltraFocus DXA system (Faxitron Bioptics, LLC) with automatic exposure control.

The macroscopic examination was performed in combination with histopathological analyses using H&E staining on formalin-fixed paraffin-embedded sections (3 µm) of tissues from 29 organs as described in www.mouseclinic.de/screens/pathology. Immunohistochemistry was carried out in a Leica Bond III (Leica Biosystems) automatic stainer. Heat-induced antigen retrieval was performed with citrate buffer (pH 6) for 30 min (AR9961; Bond TM Epitope Retrieval Solution; Leica Biosystems) in 2-μm-thick sections. For the identification of specific cells in the thymus, antibodies against CD3 (Clone SP7; ZYT-RBG024; Zytomed systems) and CD45R/B220 (Clone RA3-6B2; 550286; BD Pharmingen) were employed and the staining was detected with DAB chromogen. The slides were scanned using a Hamamatsu NanoZoomer 2.0HT digital scanner and analyzed by two independent pathologists using NDP.view2 software (Hamamatsu Photonics).

### Analysis of genetic differentiation

The genomes of the Dummerstorf mouse lines were sequenced to conduct genomic analyses that included the detection of line-specific patterns of genetic differentiation with respect to the control line FZTDU^9^. This was done by averaging the per-SNP (single nucleotide polymorphism) genetic differentiation fixation index (Fst)^90^ in 50 kilobase pairs (kbp) windows (sliding window mode with size = 50 kbp and step = 25 kbp) containing at least ten SNPs using vcftools v0.1.13^91^. Per-window Fst scores were standardized into *z*-scores, in order to evaluate the data as deviations from the genomic mean (z-Fst). The X-chromosome was standardized separately. RDD were defined as highly differentiated windows in DU6 (top 15% of the z-Fst distribution) that also displayed low genetic differentiation (bottom 25% of the z-Fst distribution) in (a) all remaining lines or (b) the rest of the lines, except DU6P. Genes overlapping these windows were detected and employed for enrichment analysis using WebGestaltR^92^. Genomic regions around genes of interest were visualized with Gviz ^93^.

### Histone acetylation analysis

Histone acetylation analysis was done as previously described in refs. ^36,37,94^. Briefly, 25 mg of the liver was used for acid extraction of histones. The resuspended samples were loaded on gels and stained overnight followed by distaining with water. Gel pieces were processed for mass spectrometry analysis using heavy labeling of d6-deuterated acetic anhydride. Histone peptides were desalted using C18-StageTips, eluted in 80% acetonitrile and 0.25% trifluoracetic acid (TFA), vacuum concentrated, reconstituted in 25 µL of 0.1% TFA, and stored at 4 °C (short) or 20 °C (long).

Desalted peptides (5 µl) were injected and separated on an Ultimate 3000 RSLCnano (Thermo Fisher) with a gradient from 3–32% acetonitrile in 0.1% formic acid over 40 min at 400 nL min^−1^ in a 25 cm analytical Aurora C18 nanocolumn (75 μm ID 120 Å, 1.6 μm, Ion Opticks). The effluent from the HPLC was directly electrosprayed into a Q-Exactive HF instrument operated in data-dependent mode to automatically switch between full-scan MS and MS/MS acquisition. Survey full-scan MS spectra (from m/z 250–1600) were acquired with resolution 60,000 at m/z 400 (automatic gain control (AGC) target of 3 × 10^6^). The ten most intense peptide ions with charge states between 2 and 5 were sequentially isolated to a target value of 1 × 10^5^ and fragmented at 27% normalized collision energy. Typical mass spectrometric conditions were: spray voltage of 1.5 kV; no sheath and auxiliary gas flow; a heated capillary temperature of 250 °C; ion selection threshold of 33,000 counts.

Peak integration was performed with Skyline (Skyline-daily 64 bit, 4.2.1.19004) using doubly o triply charged MS1 precursor ions. Peaks were selected manually, and the integrated peak values (Area) were exported as .csv file for further calculations. After peak integration, the data summarization and statistical analysis were performed in Excel and R^36^.

### Staining of triglycerides in liver tissues

Liver tissue samples embedded in Tissue Tek (Weckert, Kitzingen, Germany) were cryosectioned (8-μm-thick) using a Leica CM3050 S (Leica, Bensheim, Germany) cryostat microtome. After fixation in 4% paraformaldehyde in phosphate-buffered saline for 1 min at room temperature, the slides were stained in Oil Red O solution (1 mg ml^−1^; #A12989, Alfa Aesar, Karlsruhe, Germany) in 60% isopropanol for 10 min and then washed three times in distilled water. The slides were further stained in hematoxylin for 5 min and washed for 3 min in fresh tap water before embedding with Aquatex (Roth, Germany) and dried overnight. The staining of the triglycerides was visualized with a Nikon Microphot-Fxa microscope (Nikon Instruments Europe B.V., The Netherlands) and an image analysis system (Nikon Digital Sight, DS-L2).

### RNAseq

For RNA extraction and library preparation, 50 mg of tissues (liver) were homogenized in Trizol (Thermo Fisher; cat. no. 15596026) and processed according to the manufacturer’s instructions. RNA concentration and A_260/280_ ratio were measured with NanoDrop, followed by Bioanalyzer using RNA pico assay kit according to the manufacturer’s protocol. rRNA depletion was performed using NEBNext rRNA Depletion Kit (Human/Mouse/Rat, NEB #E6310) and library preparation for RNAseq was performed using NEBNext Ultra II Directional RNA Library Prep Kit for Illumina (NEB #E7760) following the manufacturer’s protocol. Libraries were sequenced on an Illumina HiSeq 1500 instrument at the Laboratory of Functional Genomic Analysis (LAFUGA, Gene Center Munich, LMU).

For RNAseq data analysis, read mapping of mouse tissue samples to the mouse genome (GRCm38) and counting of reads mapped to genes were performed using STAR v2.5.3a^95^ using parameters–quantMode GeneCounts and providing annotation –sjdbGTFfile Mus_musculus.GRCm38.97.gtf. Aligned reads were filtered for unmapped, multi-mapped, and ambiguous reads. Reads from histones and Y chromosomes were removed. Reads were also filtered if they had low read counts in at least two samples. Differential expression analysis was carried out using DESeq2 v1.24.0^96^ at an adjusted *p* value cut-off of 0.05. GO term analysis was performed using ClusterProfiler v3.12.0^97^ at a false discovery rate (FDR) of 0.05 using the Benjamini–Hochberg procedure and with a log fold change cut-off of 0.5. GO terms containing at least a minimum of ten genes were considered.

All the plots generated for RNAseq data were obtained using ggplot2 v3.2.1^98^ unless otherwise stated. For heatmaps and Venn diagram for RNAseq and proteomics data, pheatmap v1.0.12 (Kolde, R. (2013). pheatmap: Pretty Heatmaps. R package version 0.7.7. http://CRAN.R-project.org/package=pheatmap) was used respectively with genes or proteins passing the adjusted *p* value significance of 0.05.

### Proteomics

The proteome protocol was adapted from refs. ^94,99^ with the following modifications. A total of 200 mg of frozen mice liver was homogenized in 500 µl lysis buffer (50 mM Tris-HCl pH 7.5, 500 mM NaCl, 1 ml EDTA, 0.1% NP-40 and 20% glycerol, 15 mM sodium butyrate, 60 mM of sirtinol and one protease inhibitor tablet by Roche) and then supplemented with 200 µl 6 M urea, 2 M thiourea, and 900 µl lysis buffer. To reduce disulfide bonds, samples were treated with 1 mM dithiothreitol for 45 min at 4 °C, followed by treatment with 550 mM indole-3-acetic acid for 30 min at 4 °C in the dark. About 1 M ammonium bicarbonate (Ambic) was added to the samples to get a final concentration of 1 M urea. The proteins were digested for 5 h with Lys-C (Wako) at room temperature and overnight with trypsin (Worthington). Samples were acidified and diluted with TFA to a final concentration of 1% TFA before being loaded on the Sep-Pak Light C18 cartridges (Waters). Columns were washed with 0.1% TFA and eluted with 60% acetonitrile (ACN) with 0.25% TFA. The eluates were dried out by a speed vacuum. The pellets were re-dissolved in buffer (50 mM Hepes pH 8.0 and 50 mM NaCl) and the protein concentration was measured by Nanodrop.

LC-MS/MS measurements were performed on an Ultimate 3000 RSLCnano system coupled to a Q-Exactive HF-X mass spectrometer (Thermo Fisher Scientific). For full proteome analyses, ~0.25 µg of peptides were delivered to a trap column (ReproSil-pur C18-AQ, 5 μm, Dr. Maisch, 20 mm × 75 μm, self-packed) at a flow rate of 5 μL min^−1^ in 100% solvent A (0.1% formic acid in HPLC grade water). After 10 min of loading, peptides were transferred to an analytical column (ReproSil Gold C18-AQ, 3 μm, Dr. Maisch, 450 mm × 75 μm, self-packed) and separated using a 110 min gradient from 4 to 32% of solvent B (0.1% formic acid in acetonitrile and 5% (v/v) dimethyl sulfoxide (DMSO)) at 300 nL min^−1^ flow rate. Both nanoLC solvents (solvent A = 0.1% formic acid in HPLC grade water and 5% (v/v) DMSO) contained 5% DMSO to boost MS intensity.

The Q-Exactive HF-X mass spectrometer was operated in the data-dependent acquisition and positive ionization mode. MS1 spectra (360–1300 m/z) were recorded at a resolution of 60,000 using an AGC target value of 3e6 and a maximum injection time of 45 msec. Up to 18 peptide precursors were selected for fragmentation in the case of the full proteome analyses. Only precursors with charge states 2 to 6 were selected and dynamic exclusion of 30 s was enabled. Peptide fragmentation was performed using higher energy collision-induced dissociation and normalized collision energy of 26%. The precursor isolation window width was set to 1.3 m/z. MS2 resolution was 15,000 with (AGC) target value of 1e^5^ and a maximum injection time of 25 ms (full proteome).

Peptide identification and quantification were performed using MaxQuant (version 1.6.3.4) with its built-in search engine Andromeda^100,101^. MS2 spectra were searched against the Uniprot *Mus musculus* proteome database (UP000000589, 54,208 protein entries, downloaded 22.3.2019) supplemented with common contaminants (built-in option in MaxQuant). Trypsin/P was specified as the proteolytic enzyme. The precursor tolerance was set to 4.5 ppm, and fragment ion tolerance to 20 ppm. Results were adjusted to 1% FDR on peptide spectrum match level and protein level employing a target-decoy approach using reversed protein sequences. The minimal peptide length was defined as seven amino acids, the “match-between-run” function was disabled. For full proteome analyses, carbamidomethylated cysteine was set as fixed modification and oxidation of methionine and N-terminal protein acetylation as variable modifications.

For the statistical analysis of the proteomics data, six biological replicates were measured in young and old Titan as well as young and old control mice. Protein abundances were calculated using the LFQ (label-free quantification) algorithm from MaxQuant^102^. Before further downstream analyses, protein LFQ values were logarithm (base 10) transformed. Next, Limma^103^ was used to identify the differentially expressed proteins between young control vs. young Titan mice; young control vs. old control mice; young Titan vs. old Titan mice, and old control vs. old Titan mice. The resulting *p* values were adjusted by the Benjamini–Hochberg algorithm^104^ to control the FDR. The differential analyses were performed on proteins that were identified in at least four out of six biological replicate samples in both groups under comparison.

Gene set annotations were downloaded from MSigDB^105^, including the GO annotation (C5 category) and pathway annotation (C2 category). The gene IDs of differentially expressed proteins were mapped to the gene set annotations. The significance of over-representation was evaluated using Fisher’s exact test.

### Total RNA extraction

RNA extraction was prepared from 30 mg of deep-frozen liver tissue. The tissue was homogenized in 1 mL TRItidy G™ (Applichem) via pestle and incubated at room temperature for 5 min After the addition of 200 µL chloroform the sample was mixed 15 s with the Vortex and again incubated for 2 min at room temperature with the following centrifugation step with 12,000 × *g* at 4 °C for 15 min The aqueous phase was filled in a new vial and mixed with 500 µL −20 °C isopropanol and incubated 10 min at −20 °C. For pelletization, the sample was centrifuged at 8000 × *g*, 4 °C for 10 min and the supernatant was discarded. Two wash steps with 1 mL of 70% ethanol at −20 °C were followed. The clean pellet was air-dried and dissolved in 100 µL nuclease-free water. RNA concentration was measured with NanoDrop 2000.

### Quantitative real-time PCR for male mice

About 800 ng RNA was taken for cDNA synthesis. The cDNA was generated using the SensiFAST™cDNA Synthesis Kit (Bioline). For the PCR reaction, the SensiFAST™SYBR^®^ No-Rox Kit (Bioline) was used. The cDNA amount in all reactions was 1.25 ng and the primer concentration was 4 pmol/µL. RT-PCR was performed using the Roche Lightcycler 96. The annealing temperature was 60 °C. The RT-PCR results were normalized to the levels of beta-actin. Each sample was measured using two technical replicates. Primers are listed in Supplementary Data 8.

### Quantitative real-time PCR for female mice

Prior to cDNA synthesis, 1 µg RNA was taken for DNase digestion. The digestion was performed according to the supplier guidelines (Thermo Scientific™ DNase I). For cDNA synthesis 1 µg DNase digested RNA was taken. The cDNA was generated using the SensiFAST™cDNA Synthesis Kit (Bioline). For the PCR reaction, the SensiFAST™SYBR^®^ No-Rox Kit (Bioline) was used. The cDNA concentration in all reactions was 1.25 ng and the primer concentration was 4 pmol/µL. RT-PCR was performed using the Roche Lightcycler 96. The annealing temperature was 60 °C. The RT-PCR results were normalized to the levels of beta-actin. Each sample was measured using two technical replicates.

### Microbiome analysis

The intestinal microbiome analysis comprised 20 females and 12 males from the DU6/Titan strain equally balanced for SBF and ERF. The feeding trial lasted from week 12 of age until slaughter at week 20 for the females and week 18 for the males. DNA extraction from 200 mg cecum digesta samples was performed using the DNeasy PowerLyzer PowerSoil Kit (QIAGEN, Hilden, Germany), including additional incubation steps at 70 °C and 95 °C for 10 min each and bead beating with the Precellys 24 Homogenizer (PEQLab Biotechnology GmbH, Darmstadt, Germany) to improve sample breakdown. After quantifying the DNA concentration with the Nanodrop 2000 for each sample, 25 ng of DNA were used to individually amplify the hypervariable region V4 of the 16S rRNA gene in duplicate by PCR. Therefore, primers were designed to contain 16S-specific sequences, 515´F (GTGBCAGCMGCCGCGGTAA) and 806R (GGACTACHVGGGTWTCTAAT), flow cell adapter sequences, and unique index sequences as previously described in refs. ^106,107^. The pooled replicates were purified using the SequalPrep normalization plate (Thermo Fisher Scientific, Darmstadt, Germany) and then combined in equal proportions. The amplicons were sequenced with the HiSeq 2500 (Illumina, San Diego, CA) in overlapping 250 bp reads. After the sequencing data were de-multiplexed according to the sample-specific index combinations, the mothur tool (version 1.47) was used for further processing^108^. The Silva database (release 138; https://www.arb-silva.de/) was used for sequence alignment, classification, and finally, annotation of operational taxonomic units (OTU) derived from clustering of sequences with ≥97% identity. Statistical analyses were performed with the R package DESeq2 to identify microbial taxa with varying abundance between the differently fed Titan mice^96^. For this purpose, very low abundant taxa were removed, thus keeping those that had at least 50 read counts in at least half of the samples. Using a negative binomial Wald test, the analysis was conducted for each sex separately with dietary treatment included as a fixed effect in the model. Differences at a Benjamini–Hochberg adjusted *p* value <0.05 were considered significant.

### Statistics and reproducibility

Unless stated otherwise in the RNAseq and proteomics experimental method, statistics and graphing were conducted on GraphPad Prism 9. In general, we test for normality prior to comparing with the *t-*test of MWU. Unpaired two‐tailed *t*‐tests with Welch’s correction (parametric) or MWU testing (non-parametric) were used for calculating the *p* values unless stated otherwise. For example, we used paired testing in Fig. 6 as siblings were compared. A multiple correction (FDR) was performed for SBF vs. ERF RT-PCR and plasma analysis. A *p* value ≤0.05 was used as the level of significance. Of note, no FDR was done for histone 4 analysis as all the modifications are calculated from a single ion.

Importantly, according to the GMC analysis outline, correction for multiple testing was not performed when analyzing the GMC dataset (See GMC website).

### Reporting Summary

Further information on research design is available in the Nature Research Reporting Summary linked to this article.

## Supplementary information

Supplementary information

Description of Additional Supplementary Files

Supplementary data 1

Supplementary data 2

Supplementary data 3

Supplementary data 4

Supplementary data 5

Supplementary data 6

Supplementary data 7

Supplementary data 8

Reporting Summary

## Data Availability

The proteomic raw data and MaxQuant search files have been deposited in the ProteomeXchange Consortium (http://proteomecentral.proteomexchange.org) via the PRIDE partner repository and can be accessed using the dataset identifier PXD019030. All RNAseq data have been deposited in GEO (https://www.ncbi.nlm.nih.gov/geo/) and are accessible under the GEO accession ID GSE150073.
