# Supplementary material for: Dietary intervention improves health metrics and life expectancy of the genetically obese Titan mouse
Source: Commun Biol. 2022 May 3;5:408. doi: 10.1038/s42003-022-03339-3 (PMC9065075; doi:10.1038/s42003-022-03339-3)
Supplement: Supplementary file 1 — Supplementary information [file 42003_2022_3339_MOESM1_ESM.pdf]

Supplementary figures and tables

Dietary intervention improves health metrics and life expectancy of the genetically obese Titan mouse

Annika Müller-Eigner[1†], Adrián Sanz-Moreno[2†], Irene de-Diego[1†], Anuroop Venkateswaran Venkatasubramani[3], Martina Langhammer[4], Raffaele Gerlini[2,5], Birgit Rathkolb[2,5,6], Antonio Aguilar-Pimentel[2], Tanja Klein-Rodewald[2], Julia Calzada-Wack[2], Lore Becker[2], Sergio Palma-Vera[7], Benedikt Gille[1], Ignasi Forne[3], Axel Imhof[3], Chen Meng[8], Christina Ludwig[8], Franziska Koch[9], John T. Heiker[10], Angela Kuhla[11], Vanessa Caton[12], Julia Brenmoehl[12], Henry Reyer[12], Jennifer Schoen[7,13], Helmut Fuchs[2], Valerie Gailus-Durner[2], Andreas Hoeflich[12], Martin Hrabe de Angelis[2,5,14] and Shahaf Peleg[1,15]

[1]Research Group Epigenetics, Metabolism and Longevity, Leibniz Institute for Farm Animal Biology, 18196 Dummerstorf, Germany
[2]Institute of Experimental Genetics, German Mouse Clinic, Helmholtz Zentrum München, German Research Center for Environment and Health (GmbH), 85764 Neuherberg, Germany
[3]Department of Molecular Biology, Biomedical Center Munich, Ludwig-Maximilians University, 82152 Planegg-Martinsried, Germany
[4]Institute Genetics and Biometry, Lab Animal Facility, Leibniz Institute for Farm Animal Biology, 18196 Dummerstorf, Germany
[5]German Center for Diabetes Research (DZD), 85764, Neuherberg, Germany
[6]Institute of Molecular Animal Breeding and Biotechnology, Gene Center, Ludwig-Maximilians-University Munich, 81377 Munich, Germany
[7]Institute of Reproductive Biology, Leibniz Institute for Farm Animal Biology, FBN, 18196 Dummerstorf, Germany.
[8]Bavarian Center for Biomolecular Mass Spectrometry (BayBioMS), Technical University of Munich, 85354, Freising, Germany
[9]Institute of Nutritional Physiology, Leibniz Institute for Farm Animal Biology, 18196 Dummerstorf, Germany
[10]Helmholtz Institute for Metabolic, Obesity and Vascular Research (HI-MAG) of the Helmholtz Zentrum München at the University of Leipzig and University Hospital Leipzig, Leipzig, Germany
[11]Institute for Experimental Surgery, Rostock University Medical Center, Rostock, Germany
[12]Institute for Genome Biology, Leibniz Institute for Farm Animal Biology, 18196 Dummerstorf, Germany
[13]Department of Reproduction Biology, Leibniz Institute for Zoo and Wildlife Research (IZW), Berlin, Germany
[14]Chair of Experimental Genetics, TUM School of Life Sciences (SoLS), Technische Universität München, 85354 Freising, Germany
[15]Institute of Neuroregeneration and Neurorehabilitation of Qingdao University, Qingdao, China

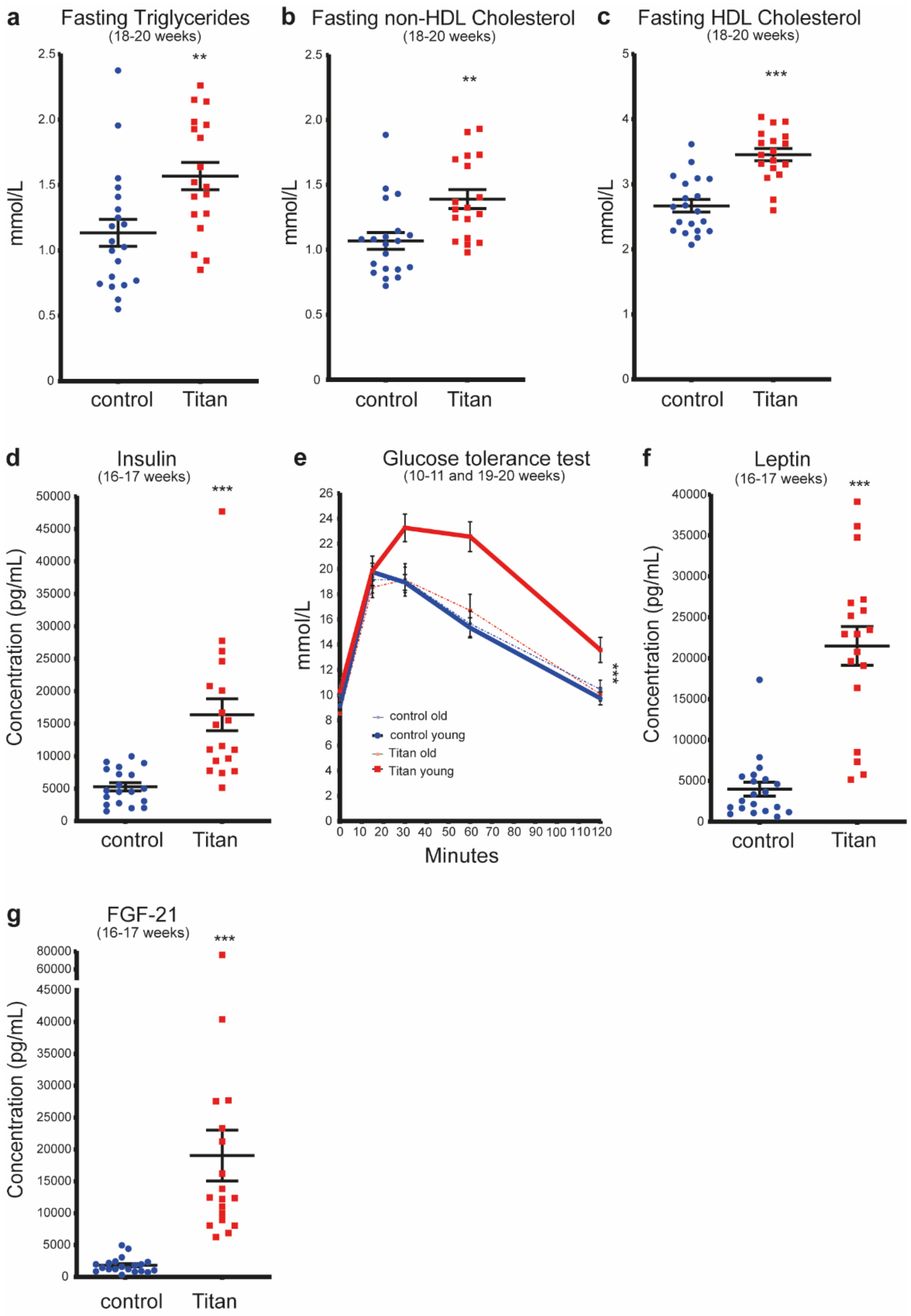

Supplementary Figure 1: Titan mice display several molecular criteria for metabolically unhealthy obesity.

a-c Plasma analyses comparing control and Titan mice after fasting at 18–20 weeks. Titan mice showed higher levels of triglycerides (p = 0.0055) and both non-HDL (high-density lipoprotein) (p = 0.0022) and HDL cholesterol in control (n = 20) and Titan (n = 19 Titan). **d** Insulin levels in control (n = 19) and Titan (n = 18) mice at 16–17 weeks of age. **e** Glucose tolerance test at 10-11 weeks of age in control (n = 20) and Titan (n = 17) and 16-17 weeks of age in control (n = 18) and Titan (n = 14) animals. 10-11 weeks old Titan mice show impaired glucose clearance ($p < 0.0001$, area under curve) compared to the control group. In contrast, no significant difference (Mann-Whitney U test, p = 0.8077, area under curve) is observed at 16-17 weeks of age. **f** Leptin and **g** FGF21 levels in control (n = 20) and Titan (n = 18) mice. **$p < 0.01$ ***$p < 0.001$. Error bars indicate SEM. Unpaired two-tailed t-tests with Welch's correction were used to calculate p-values.

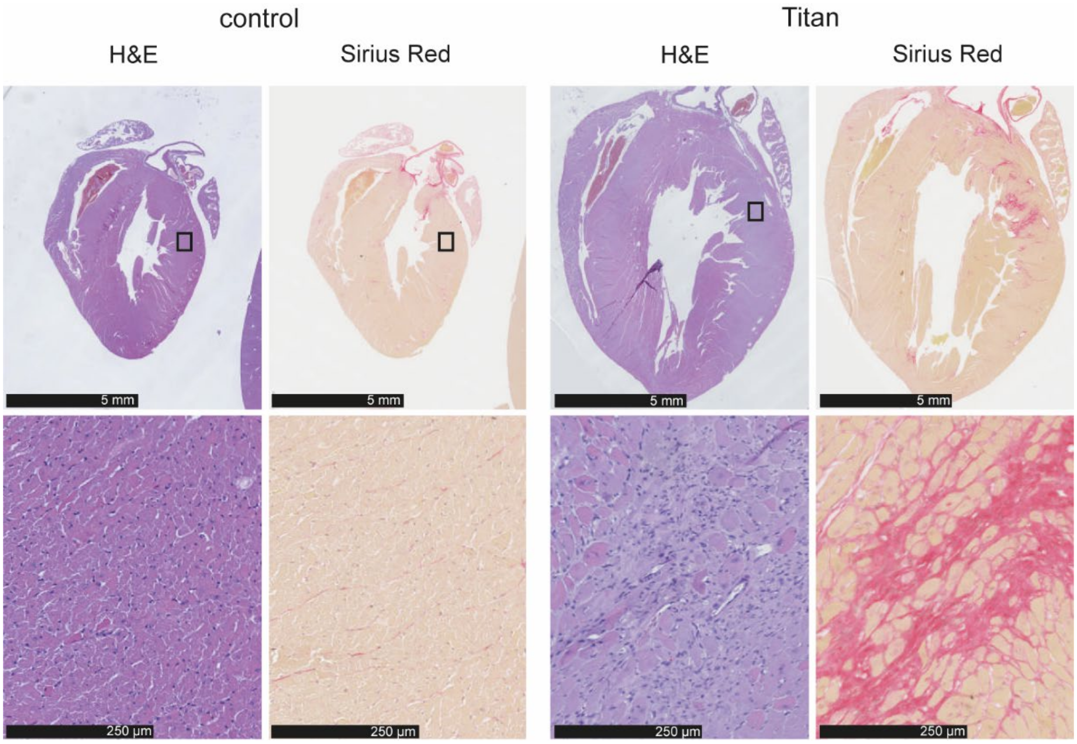

Supplementary Figure 2: Titan mice show signs of heart fibrosis.
Hematoxylin and eosin and Sirius Red stainings of the heart from 16-17-weeks-old control and Titan mice are shown (0.45x magnification). Sirius Red staining allows a better visualization of fibrotic tissue. Higher magnification pictures from rectangles were taken at 10x magnification.

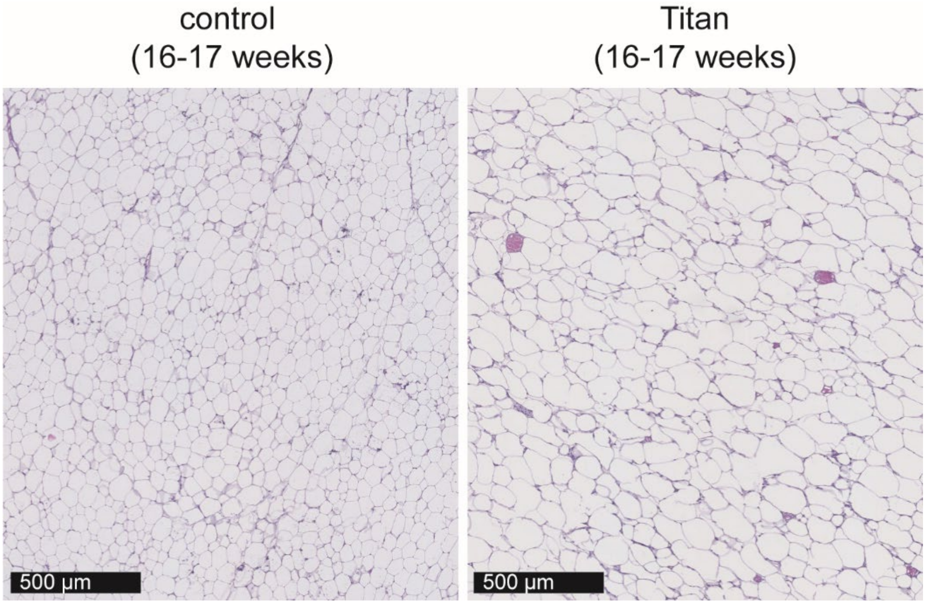

Supplementary Figure 3: WAT H&E staining comparison of control vs. Titan mice.
Hematoxylin and eosin staining of perigonadal white adipose tissue shows the hypertrophic size of adipocytes in 16–17 week old Titan mice (5x magnification).

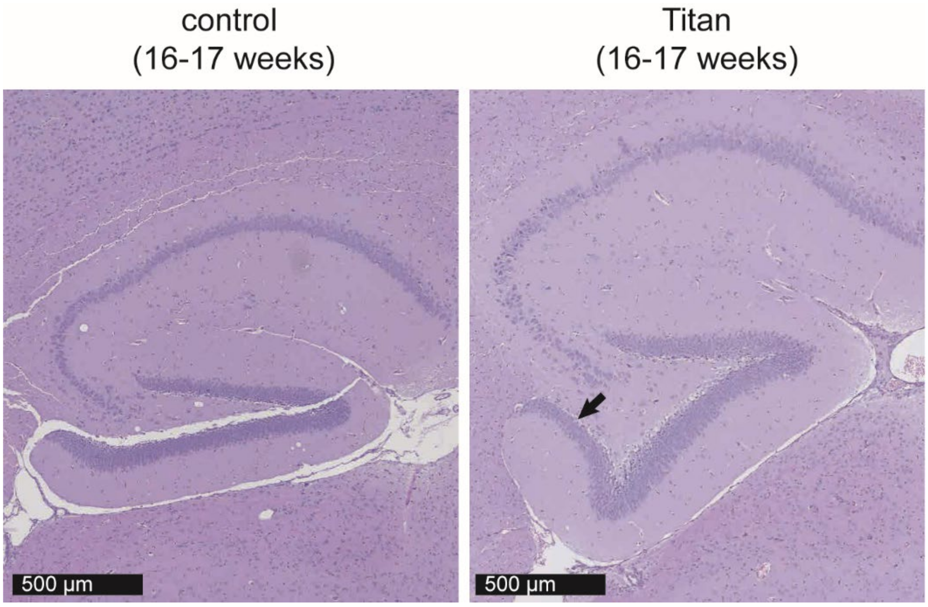

Supplementary Figure 4: Hippocampal H&E staining comparison of control vs. Titan mice. Hematoxylin and eosin staining of the hippocampus shows an abnormal angle of the dentate gyrus (arrow) in 16–17 weeks of age Titan mice (5x magnification).

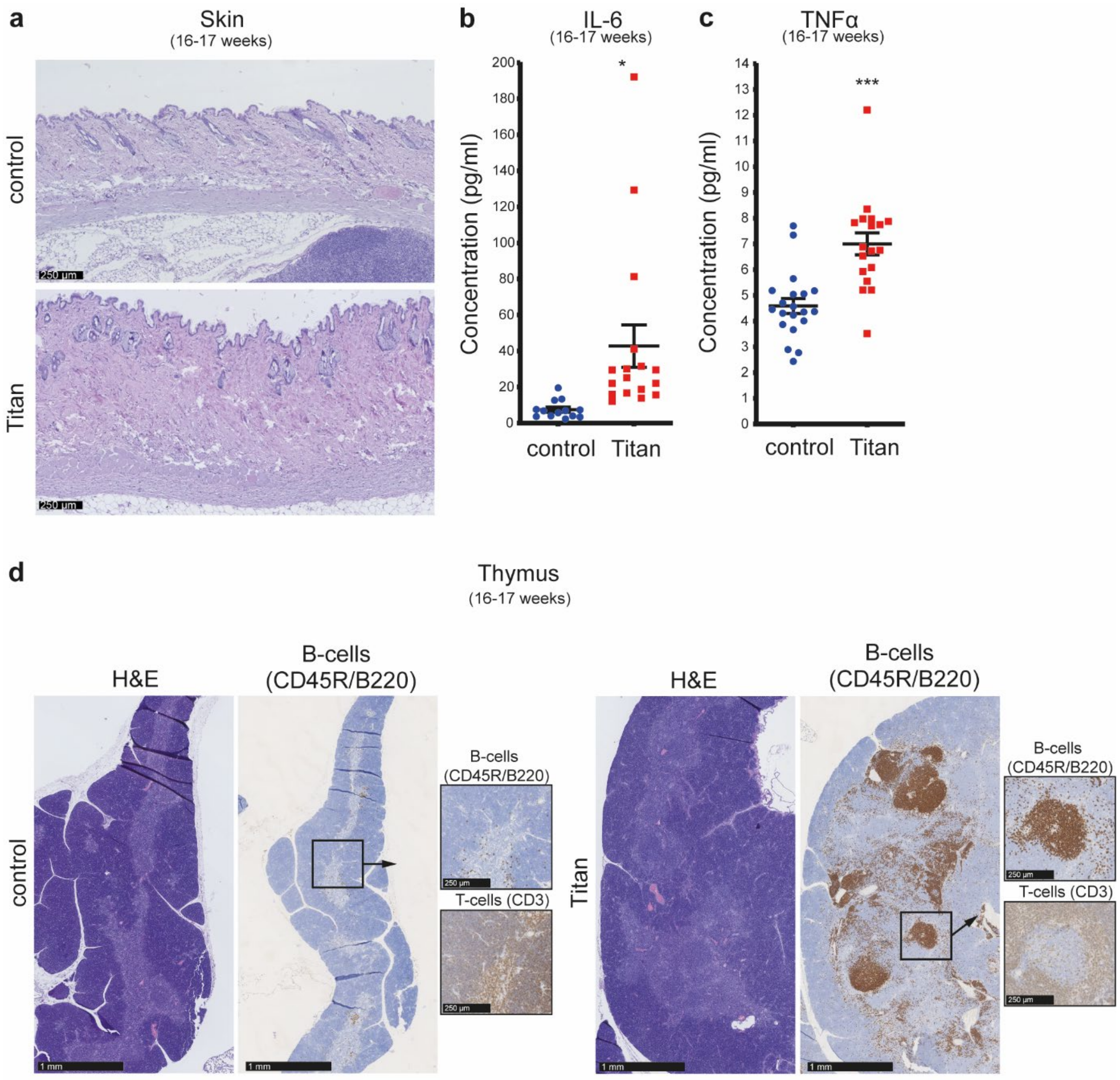

Supplementary Figure 5: Titan mice show thickened dermis and early signs of increased inflammation.

Comparison of Titan and control mice at 16–17 weeks of age. (n = 4–6 per group). **a** Representative images of hematoxylin and eosin staining of the skin. The dermis of Titan mice is substantially thicker compared to control animals **b,c** IL-6 plasma levels (p = 0.0086) in control (n = 13) and Titan (n = 17) mice, and TNFα plasma levels in control (n = 20) and Titan (n = 18) mice. **d** Representative images of hematoxylin and eosin staining and B-cell immunohistochemistry (IHC) of the thymus of control (left) and Titan (right) mice (2.5x magnification). IHC of thymic medullar nodes revealed that they are composed mainly of B-cells (CD45R/B220-positive) instead of T-cells (CD3-positive). Images in squares were taken at 10x magnification. *p < 0.05, ***p < 0.001. Error bars indicate SEM. Unpaired two-tailed t-tests with Welch's correction were used to calculate p-values.

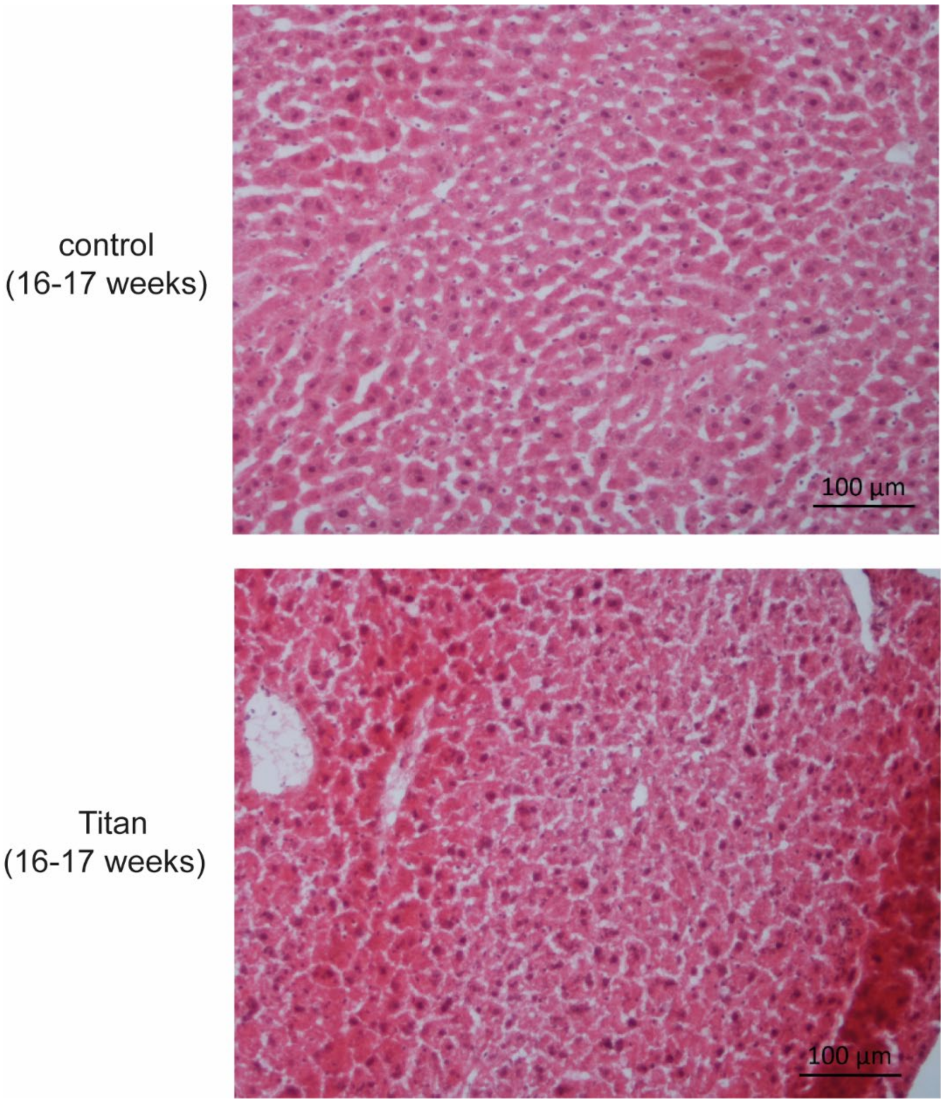

Supplementary Figure 6: Liver H&E staining comparison of control vs. Titan mice.
Shown are representative hematoxylin and eosin-stained images of the liver from 16-17-weeks old control and Titan mice.

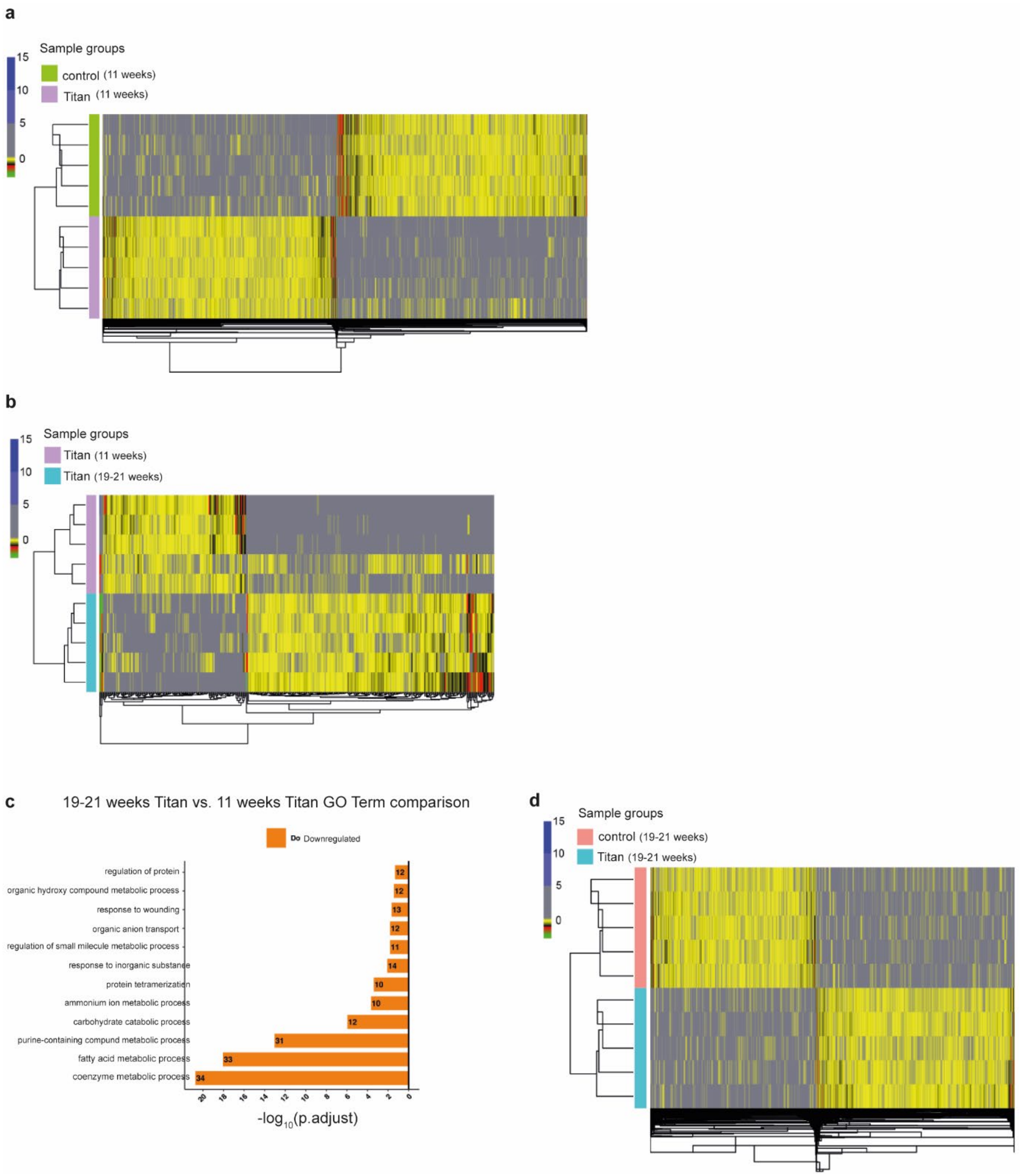

Supplementary Figure 7: Transcriptome changes in the liver of younger vs. older Titan mice.
a Heat map showing significantly altered genes between 11-week-old control and Titan mice. **b** Heat map showing significantly altered genes between 11- and 19-21-week-old Titan mice. **c** Gene ontology term analysis of altered genes (b) revealed a decrease in various metabolic processes including fatty acid metabolism. **d** Heat map showing significantly altered genes between 19-21-week-old control and Titan mice (n = 5 per group).

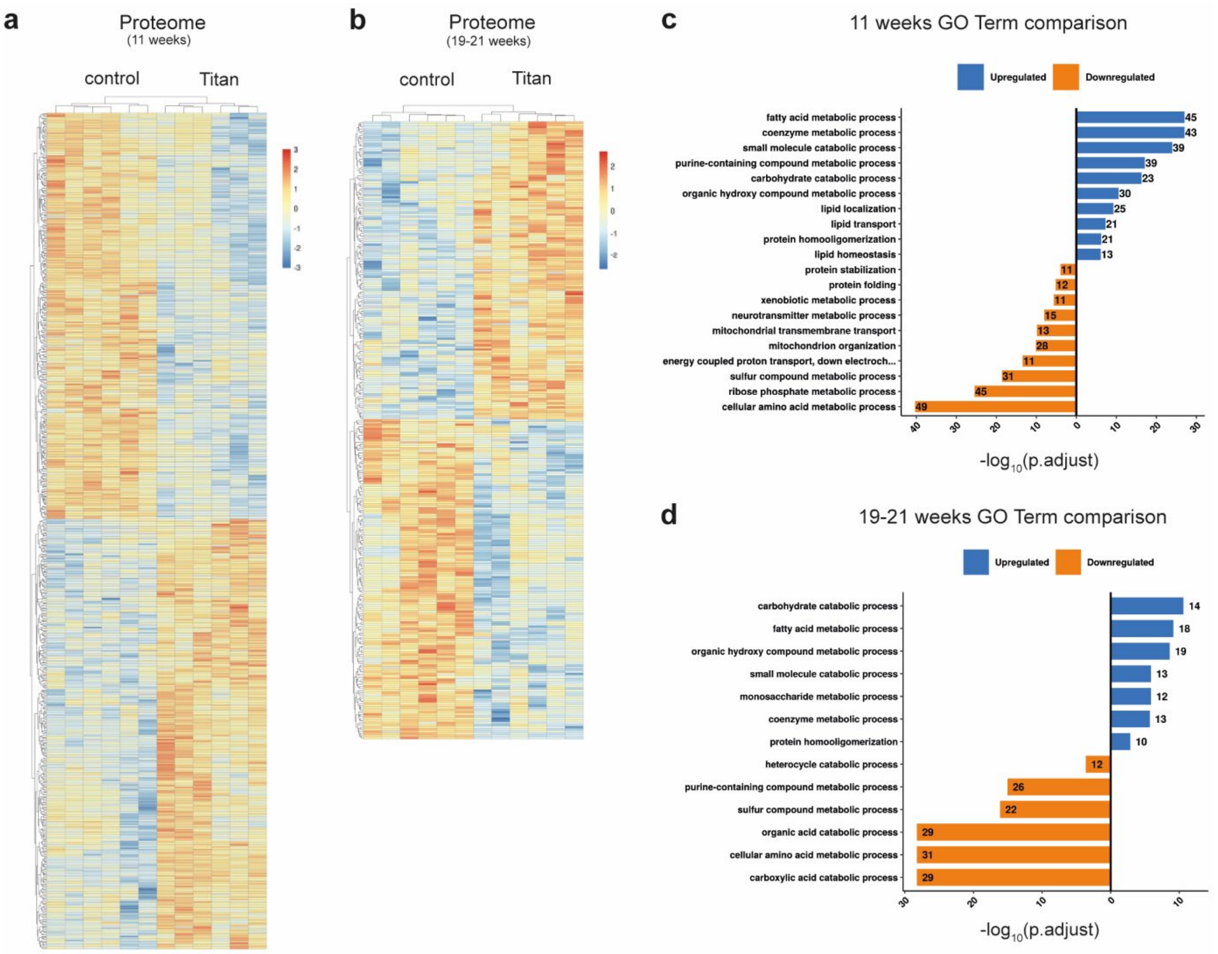

Supplementary Figure 8: Liver proteome analyses reveal differences in metabolic protein levels between control and Titan mice.

a Heat map comparing proteomes of 11-week-old control and Titan mice. **b** Heat map comparing proteomes of 19-21-week-old control and Titan mice. **c** Gene ontology (GO) term analysis of 11-week-old mice indicated increased fatty acid metabolism, various lipid catabolism processes, and coenzyme metabolism while downregulation in xenobiotic, amino acid and sulfur metabolism. **d** GO term analysis of 19-21-week-old mice showed upregulation of proteins involved in fatty acid metabolism, carbohydrate catabolism and coenzyme metabolism and downregulation of proteins involved in amino acid and sulfur metabolism (n = 6 per group).

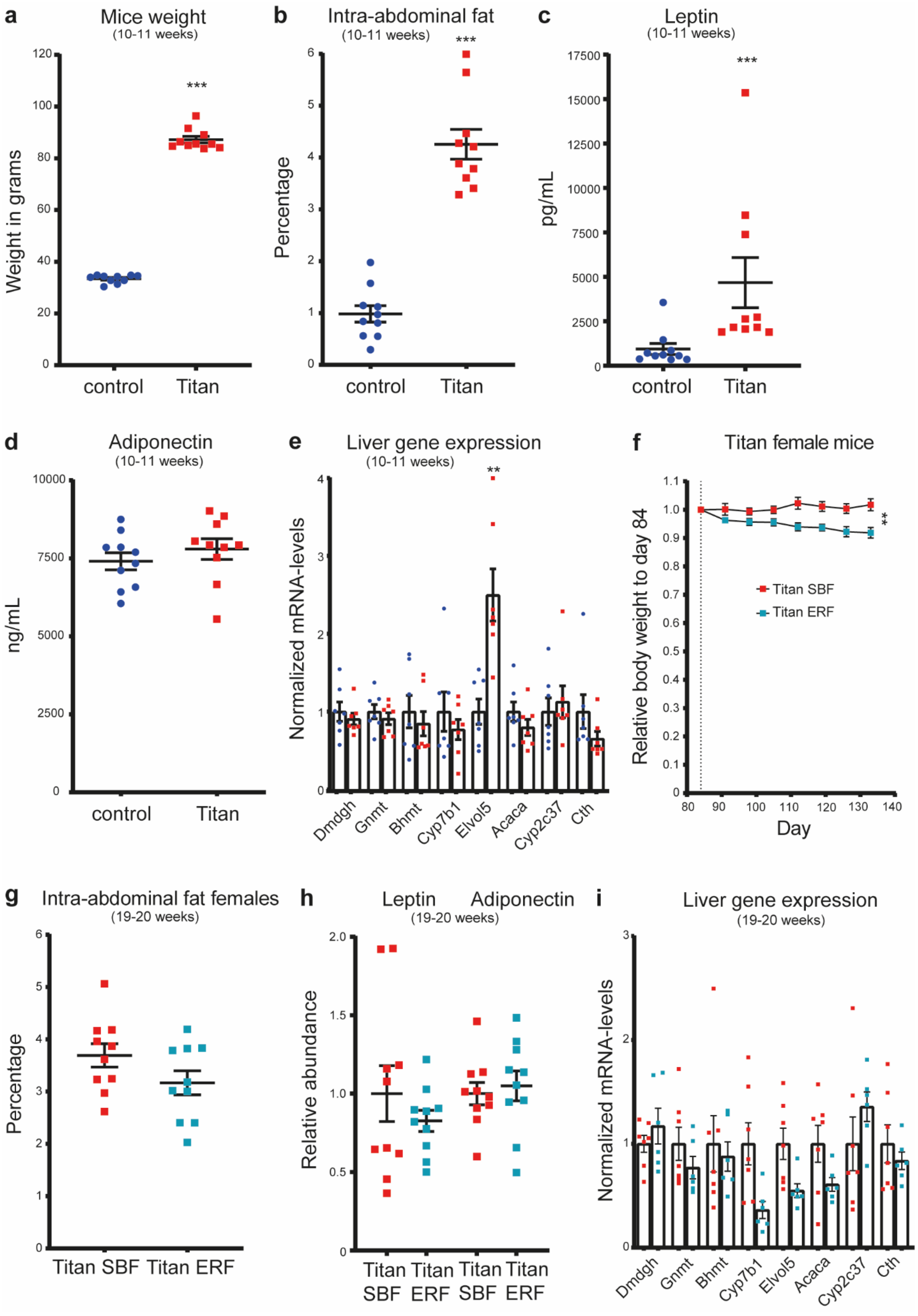

Supplementary Figure 9: Characterization of female Titan mice.

a At 10–11-weeks old, Titan female mice reached 87 grams on average, whereas average control female animals weighed 33 grams (*n* = 10 per group). Mann-Whitney *U*-test (MWU), $p < 0.0001$. **b** Percentage of intra-abdominal fat (*n* = 10 per group) at the age of 10–11-weeks. $p < 0.0001$. **c** Similarly

to males, 10–11-weeks old Titan female mice show increased leptin levels compared with female control mice (*n* = 10 per group). MWU-test, $p = 0.0005$. **d** Similar adiponectin levels were observed between 10–11-weeks Titan and control female mice (*n* = 10 per group). p = 0.3756. **e** RT-PCR comparing gene expression of candidate genes of 10-11 weeks of age Titan and control female mice (*n* = 7 per group). Non-parametric test was performed followed by multiple corrections to calculate *p*-values; pval.adj (*Dmdg*) = 0.992, (*Gnmt*) = 0.992, (*Bhmt*) = 0.992, (*Cyp7b1*) = 0.992, (*Elovl5*) = 0.00928, (*Cyp2c37*) = 0.992, (*Acaca*) = 0.992, (*Cth*) = 0.299. **f** Switching standard breeding feed (SBF) to enegery reduced feed (ERF) at 12 weeks resulted in a persistent average weight loss in Titan females (*n* = 10 per group). MWU-test at 19 weeks, $p = 0.0015$. **g,h** ERF- and SBF-fed female Titan mice have similar percentage of intra-abdominal fat at 19-20 weeks ($p = 0.121$), leptin ($p = 0.3746$) and adiponectin ($p = 0.6817$; *n* = 10 per group). **i** RT-PCR comparing gene expression of candidate genes of ERF- and SBF-fed Titan mice females at 19-20 weeks of age (*n* = 7 per group). *t*-test was performed followed by multiple correction to calculate *p*-values; pval.adj (*Dmdg*) = 0.948, (*Gnmt*) = 0.588, (*Bhmt*) = 0.972, (*Cyp7b1*) = 0.0938, (*Elovl5*) = 0.0634, (*Cyp2c37*) = 0.588, (*Acaca*) = 0.588, (*Cth*) = 0.974. **$p < 0.01$, ***$p < 0.001$.

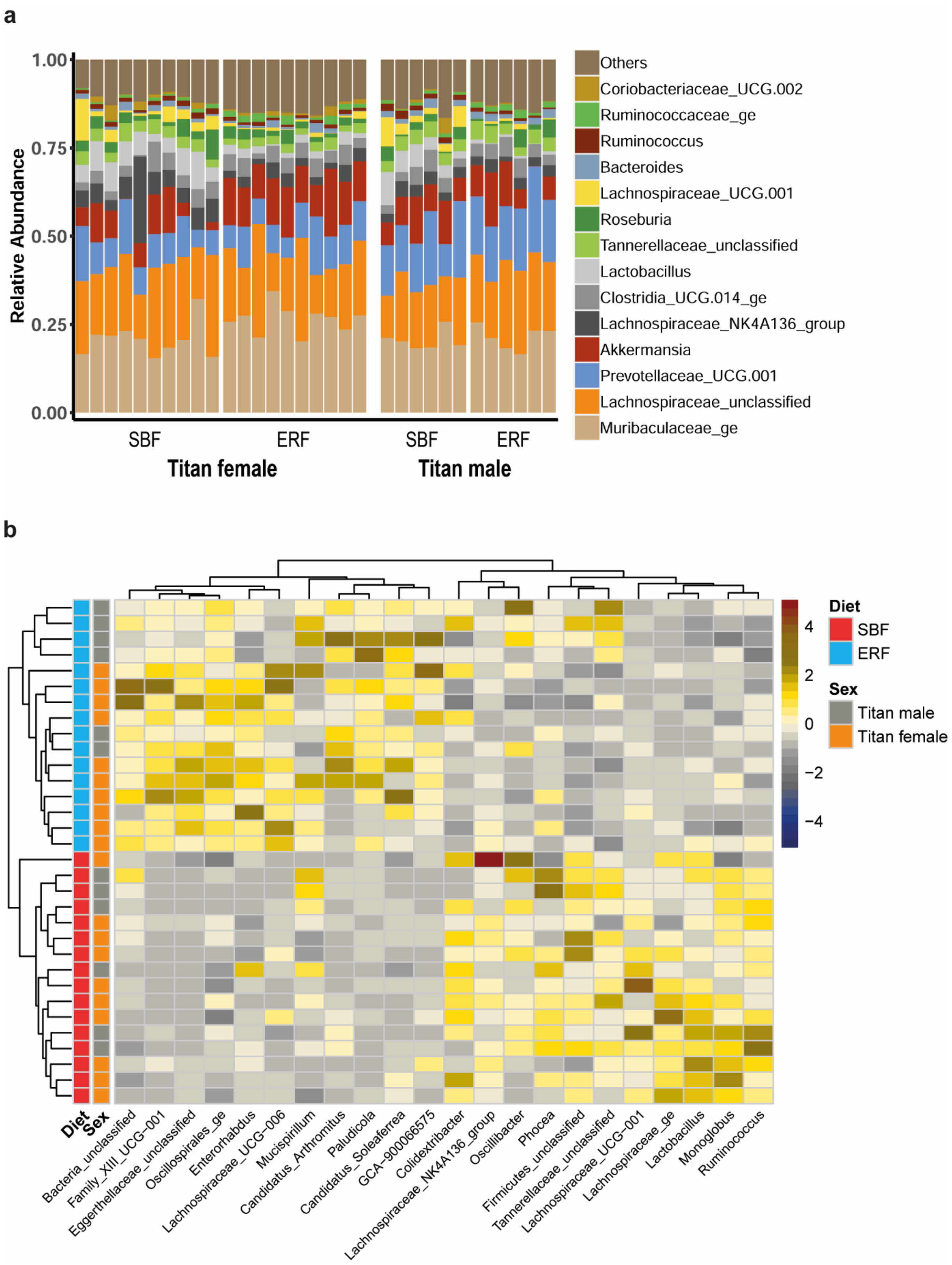

Supplementary Figure 10: Late dietary intervention by switching to ERF alters the microbiome in Titan mice.

a Taxa plot representation of the microbial composition considering the most abundant genera in caecum digesta of standard breeding feed (SBF) and energy reduced feed (ERF) fed Titan mice. **b** Heat map showing significantly changed microbial genera between ERF- and SBF-fed Titan mice.

Supplementary Tables

Supplementary table 1: Lifespans in days for Titan mice receiving standard breeding feed (SBF) and energy reduced feed (ERF), respectively

Survival [Days]	Titan SBF	Titan ERF
**Minimum**	68	71
**10% Percentile**	123	214.8
**25% Percentile**	231.5	264
**75% Percentile**	405	447
**90% Percentile**	475	504
**Maximum**	614	660
**Median**	323.5	374
**Mean**	317.4	359.5
**Std. Error**	11.49	11.45

Supplementary table 2: Diet composition

Nutrients of the standard breeding feed (SBF) and energy reduced feed (ERF) used in the dietary intervention trial. The lower energy density of the ERF is largely achieved by a higher proportion of dietary fiber. Micronutrients not listed can be considered equal in both feeds. ssniff Spezialdiäten GmbH, Soest, Germany, V1124-300 (SBF) and V1574-300 (ERF), autoclavable.

	**Standard breeding feed**	**Energy reduced feed**
**Metabolizable energy [MJ kg^{-1}]**	14.0	11.7
**Crude protein [%]**	22.0	15.0
**Crude fat [%]**	4.5	3.1
**Crude fiber [%]**	3.9	14.2
**Crude ash [%]**	6.5	7.8
**Starch [%]**	34.2	20.8
**Sugar [%]**	5.1	5.4
**Nitrogen free extracts [%]**	51.2	48.8
**Micronutrients (selection)**		
**Potassium [%]**	0.98	1.47
**Fatty acid C18:0 [%]**	0.14	0.08
**Fatty acid C18:1 [%]**	1.03	0.43
**Fatty acid C18:2 [%]**	2.42	1.44
**Lysine [%]**	1.48	0.84
**Methionine [%]**	0.50	0.36
**Cysteine [%]**	0.39	0.27
**Threonine [%]**	0.83	0.58
**Arginine [%]**	1.38	0.77
**Histidine [%]**	0.57	0.34
**Valine [%]**	1.06	0.75
**Isoleucine [%]**	0.95	0.60
**Leucine [%]**	1.67	1.17
**Phenylalanine [%]**	1.04	0.68
**Glycine [%]**	0.95	0.69
**Glutamic acid [%]**	4.56	2.67
**Aspartic acid [%]**	2.20	1.24
**Proline [%]**	1.38	1.06
**Serine [%]**	1.17	0.68
**Alanine [%]**	1.01	0.78
**Vitamin K [mg]**	20	80
**Iron [mg]**	192	276
**Dietary composition**	Wheat and wheat products, soybean/-oil, products, corn, minerals, yeast, micronutrient supplements	Alfalfa meal, wheat and wheat products, barley, corn gluten, sunflower seeds, oat bran, soybeans, sugar beet pulp, molasses, micronutrient supplements
