# Supplementary material for: Dietary intervention improves health metrics and life expectancy of the genetically obese Titan mouse
Source: Commun Biol. 2022 May 3;5:408. doi: 10.1038/s42003-022-03339-3 (PMC9065075; doi:10.1038/s42003-022-03339-3)
Supplement: Supplementary file 2 — Description of Additional Supplementary Files [file 42003_2022_3339_MOESM2_ESM.pdf]

Description of Additional Supplementary Files

File name: Supplementary data 1
Description: Unique RDD in Titan mice

File name: Supplementary data 2:
Description: Unique RDD in Titan mice excluding DU6P mice

File name: Supplementary data 3
Description: RNAseq significantly altered genes, 11 weeks

File name: Supplementary data 4
Description: RNAseq significantly altered genes, 19-21 weeks

File name: Supplementary data 5
Description: Proteome data for supplementary figure 8

File name: Supplementary data 6
Description: Raw data for plasma analysis for figure 6c

File name: Supplementary data 7
Description: Microbiome analysis of SBF vs ERF male and female mice

File name: Supplementary data 8
Description: Key resources and real time PCR primer sequences
