# Supplementary material for: Dietary intervention improves health metrics and life expectancy of the genetically obese Titan mouse
Source: Commun Biol. 2022 May 3;5:408. doi: 10.1038/s42003-022-03339-3 (PMC9065075; doi:10.1038/s42003-022-03339-3)
Supplement: Supplementary file 11 — Reporting Summary [file 42003_2022_3339_MOESM11_ESM.pdf]

nature research

Corresponding author(s): Shahaf Peleg

Last updated by author(s): Mar 24, 2022

Reporting Summary

Nature Research wishes to improve the reproducibility of the work that we publish. This form provides structure for consistency and transparency in reporting. For further information on Nature Research policies, see our Editorial Policies and the Editorial Policy Checklist.

Statistics

For all statistical analyses, confirm that the following items are present in the figure legend, table legend, main text, or Methods section.

n/a	Confirmed	
☐	☒	The exact sample size (*n*) for each experimental group/condition, given as a discrete number and unit of measurement
☐	☒	A statement on whether measurements were taken from distinct samples or whether the same sample was measured repeatedly
☐	☒	The statistical test(s) used AND whether they are one- or two-sided<br>*Only common tests should be described solely by name; describe more complex techniques in the Methods section.*
☐	☒	A description of all covariates tested
☐	☒	A description of any assumptions or corrections, such as tests of normality and adjustment for multiple comparisons
☐	☒	A full description of the statistical parameters including central tendency (e.g. means) or other basic estimates (e.g. regression coefficient) AND variation (e.g. standard deviation) or associated estimates of uncertainty (e.g. confidence intervals)
☐	☒	For null hypothesis testing, the test statistic (e.g. *F*, *t*, *r*) with confidence intervals, effect sizes, degrees of freedom and *P* value noted<br>*Give P values as exact values whenever suitable.*
☒	☐	For Bayesian analysis, information on the choice of priors and Markov chain Monte Carlo settings
☒	☐	For hierarchical and complex designs, identification of the appropriate level for tests and full reporting of outcomes
☒	☐	Estimates of effect sizes (e.g. Cohen's *d*, Pearson's *r*), indicating how they were calculated

Our web collection on statistics for biologists contains articles on many of the points above.

Software and code

Policy information about availability of computer code

Data collection	X-ray imaging was performed in an UltraFocus DXA system (Faxitron Bioptics, LLC) with automatic exposure control.<br>Immunohistochemistry was carried out in a Leica Bond III (Leica Biosystems) automatic stainer.<br>For the identification of specific cells in the thymus, antibodies against CD3 (Clone SP7; ZYT-RBG024; Zytomed systems) and CD45R/B220 (Clone RA3-6B2; 550286; BD Pharmingen) were employed and the staining was detected with DAB chromogen. The slides were scanned using a Hamamatsu NanoZoomer 2.0HT digital scanner and analyzed by two independent pathologists using NDP.view2 software (Hamamatsu Photonics).<br>Histone acetylation was done with Q Exactive HF while the proteomics were done with Q Exactive HF-X.<br>RNA libraries were sequenced on an Illumina HiSeq 1500 instrument<br>RT-PCR was performed using the Roche Lightcycler 96<br>The staining of the triglycerides was visualized with a Nikon Microphot-Fxa microscope (Nikon Instruments Europe B.V., The Netherlands) and an image analysis system (Nikon Digital Sight, DS-L2).<br>Microbiome smaples were sequenced with the HiSeq 2500 (Illumina, San Diego, CA)
Data analysis	Statistics and graphing were conducted on GraphPad Prism 9<br>Plasma clinical chemistry analyses were performed using an AU480 clinical chemistry analyzer (Beckman-Coulter, Germany)<br>Genes overlapping these windows were detected and employed for enrichment analysis using WebGestaltR92. Genomic regions around genes of interest were visualized with Gviz .<br>For histone acetylation, peak integration was performed with Skyline (Skyline-daily (64bit) 4.2.1.19004) following by R analysis.<br>The staining of the triglycerides was analized by an image analysis system (Nikon Digital Sight, DS-L2).<br>For RNA-seq data analysis, read mapping of mouse tissue samples to the mouse genome (GRCm38) and counting of reads mapped to genes were performed using STAR v2.5.3a95 using parameters --quantMode GeneCounts and providing annotation –sjdbGTFfile Mus_musculus.GRCm38.97.gtf. Aligned reads were filtered for unmapped, multi mapped and ambiguous reads. Reads from histones and Y chromosome were removed. Reads were also filtered if they had low read counts in at least two samples. Differential expression analysis was

carried out using DESeq2 v1.24.096 at an adjusted p-value cut-off of 0.05. GO term analysis was performed using ClusterProfiler v3.12.097 at FDR of 0.05 using Benjamini-Hochberg procedure and with a log fold change cut-off of 0.5. GO terms containing at least a minimum of 10 genes were considered.
All the plots generated for RNA sequencing data were obtained using ggplot2 v3.2.198 unless otherwise stated. For heatmaps and Venn diagram for RNA sequencing and proteomics data, pheatmap v1.0.12 (Kolde, R. (2013). pheatmap: Pretty Heatmaps. R package version 0.7.7. http://CRAN.R-project.org/package=pheatmap) were used respectively with genes (or proteins) passing the adjusted p-value significance of 0.05.

For the proteome, protein abundances were calculated using the LFQ algorithm from MaxQuant102. Before further downstream analyses, protein LFQ values were logarithm (base 10) transformed. Next, Limma103 was used to identify the differentially expressed proteins between young control vs young Titan mice; young control vs old control mice; young Titan vs old Titan mice, and old control vs old Titan mice. The resulting p-values were adjusted by the Benjamini-Hochberg algorithm104 to control the false discovery rate (FDR).

For manuscripts utilizing custom algorithms or software that are central to the research but not yet described in published literature, software must be made available to editors and reviewers. We strongly encourage code deposition in a community repository (e.g. GitHub). See the Nature Research guidelines for submitting code & software for further information.

Data

Policy information about availability of data

All manuscripts must include a data availability statement. This statement should provide the following information, where applicable:

- Accession codes, unique identifiers, or web links for publicly available datasets
- A list of figures that have associated raw data
- A description of any restrictions on data availability

The proteomic raw data and MaxQuant search files have been deposited to the ProteomeXchange Consortium (http://proteomecentral.proteomexchange.org) via the PRIDE partner repository and can be accessed using the dataset identifier PXD019030. All RNAseq data have been deposited in GEO (https://www.ncbi.nlm.nih.gov/geo/) and are accessible under the GEO accession ID GSE150073.

Field-specific reporting

Please select the one below that is the best fit for your research. If you are not sure, read the appropriate sections before making your selection.

☒ Life sciences ☐ Behavioural & social sciences ☐ Ecological, evolutionary & environmental sciences

For a reference copy of the document with all sections, see nature.com/documents/nr-reporting-summary-flat.pdf

Life sciences study design

All studies must disclose on these points even when the disclosure is negative.

Sample size

Generally, the samples size for mouse characterization in the German mouse clinic is n=15 per group (www.mouseclinic.de). However, as our mice (in particular the control mice) are non-inbred, the GMC and us have decided to increase the starting number to n=20. For hybrid weight, we used a comparable N=15 per hybrid group.
Although no other statistical methods were utilized to determine in advance the samples size, we chose the n=5 RNAseq, RTPCR n=7 and n=6-7 for proetomics studies, based on our previous studies (Peleg et al. 2016, Bux et al, 2020).
For survival curves we took into considerations other studies involving life span and diet intervention (N=40-65, for example Mitchell et al 2019). However, as our mouse are non-inbred and we estimated n=100 mice per group (60+ for hybrid, figure 3) would be needed. For impact of diet intervention on health metrics (fat, plasma), we used n=7-10 mice based on previous similar studies (Mitchell et al 2019).

Data exclusions

We excluded RT-PCR values in which the technical replicas were more than 0.5 cycles appart, and repeated that specific sample. In a few incidentes, we have removed from the graph data points which were recognized as outliers (for example a control mouse in IL-6). This graph-excluded data will be available and marked in the source files.
We did not exclude any other data points.

Replication

All attempts of replication were successful. Survival curve was repeated twice (one for current mice generation and another from previous generations). Basic traits such as weight and size were replicated every 3 months with the birth of new generation. Histone acetylation comparison was done in two similar ages (2 and 4 months). The experiments of body weight of hybrids was repeated in two separate experiments. The impact of ERF on weight and fat was repeated at least three separate times. For the RTPCR, 2 technical replicates were done for each sample.
The sample size M refers to biological replicates only

Randomization

Mice were randomly assigned to different experiments. An exception was the SBF vs ERF experiments where firstly, siblings (only for males) were selected and then randomly spilt between SBF and ERF treatment

Blinding

Due to the obvious differences in the size of the mice, it was impossible to blind the investigators between the control and Titan mice. For the omics data, the mice were given a number during the data collection and were analyzed by another investigator with linking the sample number to the mouse genotype.

Reporting for specific materials, systems and methods

We require information from authors about some types of materials, experimental systems and methods used in many studies. Here, indicate whether each material, system or method listed is relevant to your study. If you are not sure if a list item applies to your research, read the appropriate section before selecting a response.

Materials & experimental systems

n/a	Involved in the study
☐	☒ Antibodies
☒	☐ Eukaryotic cell lines
☒	☐ Palaeontology and archaeology
☐	☒ Animals and other organisms
☒	☐ Human research participants
☒	☐ Clinical data
☒	☐ Dual use research of concern

Methods

n/a	Involved in the study
☒	☐ ChIP-seq
☒	☐ Flow cytometry
☒	☐ MRI-based neuroimaging

Antibodies

Antibodies used

Antibodies against CD3 (Clone SP7; ZYT-RBG024; Zytomed systems) and CD45R/B220 (CloneRA3-6B2; 550286; BD Pharmingen)

Validation

This is a standard procedure by the German Mouse Clinic: www.mouseclinic.de/screens/pathology.

Animals and other organisms

Policy information about studies involving animals; ARRIVE guidelines recommended for reporting animal research

Laboratory animals

We used mice of an unselected strain (FZTDU) as control and a strain selected for high body ri ass (DU6/Titan), both uniquely bred at the Leibniz Institute uf Farm Animal Biology.
At the FBN, the animals were maintained in a specific pathogen-free (SPF) environment with defined hygienic conditions at a temperature of 22.5 + 0.2°C, at least 40 % humidity and a controlled light regime with a 12:12-h light-dark cycle. The mice were kept in Polysulfon-cages of 365 x 207 x 140 mm (H-Temp PSU, I ype i i L, E urostandard, Tecni plast, Germany) and had free access to pellet concentrate and water.

Wild animals

No wild animals were used in this study

Field-collected samples

No field collected samples were used in this study

Ethics oversight

All procedures were performed in accordance with our own institutional board (Animal Protection Board from the Leibniz Institute for Farm Animal Biology). At the German Mouse Clinic, mice were maintained according to the directive 2010/63/EU, German laws and GMC housing conditions (www.mouseclinic.de).

Note that full information on the approval of the study protocol must also be provided in the manuscript.
